# Green nanocomposite: fabrication, characterization, and photocatalytic application of vitamin C adduct-conjugated ZnO nanoparticles†

**DOI:** 10.1039/d2ra06575d

**Published:** 2023-03-29

**Authors:** Dana A. Kader, Srood Omer Rashid, Khalid M. Omer

**Affiliations:** a Department of Chemistry, College of Education, University of Sulaimani Kurdistan Region Iraq srood.rashid@univsul.edu.iq; b Department of Chemistry, College of Science, University of Sulaimani Kurdistan Region Iraq

## Abstract

Recently, the conjugation of metal oxide nanoparticles with organic moieties has attracted the attention of many researchers for various applications. In this research, the green and biodegradable vitamin C was employed in a facile and inexpensive procedure to synthesize the vitamin C adduct (3), which was then blended with green ZnONPs to fabricate a new composite category (ZnONPs@vitamin C adduct). The morphology and structural composition of the prepared ZnONPs and their composites were confirmed by several techniques: Fourier-transform infrared (FT-IR) spectroscopy, field-emission scanning electron microscopy (FE-SEM), UV-vis differential reflectance spectroscopy (DRS), energy dispersive X-ray (EDX) analysis, elemental mapping, X-ray diffraction (XRD) analysis, photoluminescence (PL) spectroscopy, and zeta potential measurements. The structural composition and conjugation strategies between the ZnONPs and vitamin C adduct were revealed by FT-IR spectroscopy. The experimental results for the ZnONPs showed that they possessed a nanocrystalline wurtzite structure with quasi-spherical particles with a polydisperse size ranging from 23 to 50 nm, while the particle size appeared greater in the FE-SEM images (band gap energy of 3.22 eV); after loading with the l-ascorbic acid adduct (3), the band gap energy dropped to 3.06 eV. Later, under solar light irradiation, the photocatalytic activities of both the synthesized ZnONPs@vitamin C adduct (4) and ZnONPs, including the stability, regeneration and reusability, catalyst amount, initial dye concentration, pH effect, and light source studies, were investigated in detail in the degradation of Congo red dye (CR). Furthermore, an extensive comparison between the fabricated ZnONPs, composite (4), and ZnONPs from previous studies was performed to gain insights to commercialize the catalyst (4). Under optimum conditions, the photodegradation of CR after 180 min was 54% for ZnONPs and 95% for the ZnONPs@l-ascorbic acid adduct. Moreover, the PL study confirmed the photocatalytic enhancement of the ZnONPs. The photocatalytic degradation fate was determined by LC-MS spectrometry.

## Abstract 2

The photocatalytic efficiency of ZnO NPs is dramatically improved through the conjugation with benign l-ascorbic acid adduct.

## Graphical abstract

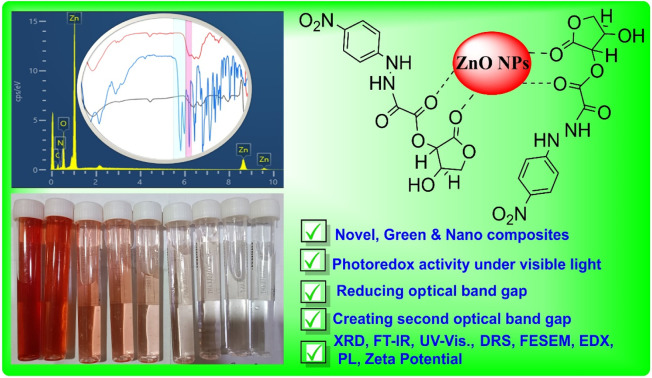

## 1. Introduction

Nowadays, nanotechnology is having a significant impact on different aspects of human life, including enhancing the quality of life and enabling the development of many innovative goods. Numerous nanoparticles (NPs) have already been fabricated with various protocols and utilized for various applications.^1^ In particular, zinc oxide nanoparticles (ZnONPs) have attracted significant biological and physical interests owing to their antibacterial, antifungal, wound-healing, UV-filtering, semiconducting, and piezoelectric properties. In addition, the catalytic and photochemical benefits of ZnONPs were well documented.^2–6^

Despite all these advantages of ZnONPs, they suffer from severe limitations, such as undesired UV absorption (equal to or less than 387 nm), due to their large band gap energy (approximately 3.2 eV)^7^ and the rapid recombination of photogenerated electron–hole pairs.^8^ These serious challenges have motivated researchers to invent novel strategies for improving ZnONPs. Recent studies have focused on improving ZnONPs through traditional metal and non-metal doping processes. Several efforts have been made to reduce the band gap energy of ZnONPs and to make it active under solar irradiation as a photocatalyst.^9–16^

**Fig. 1:**
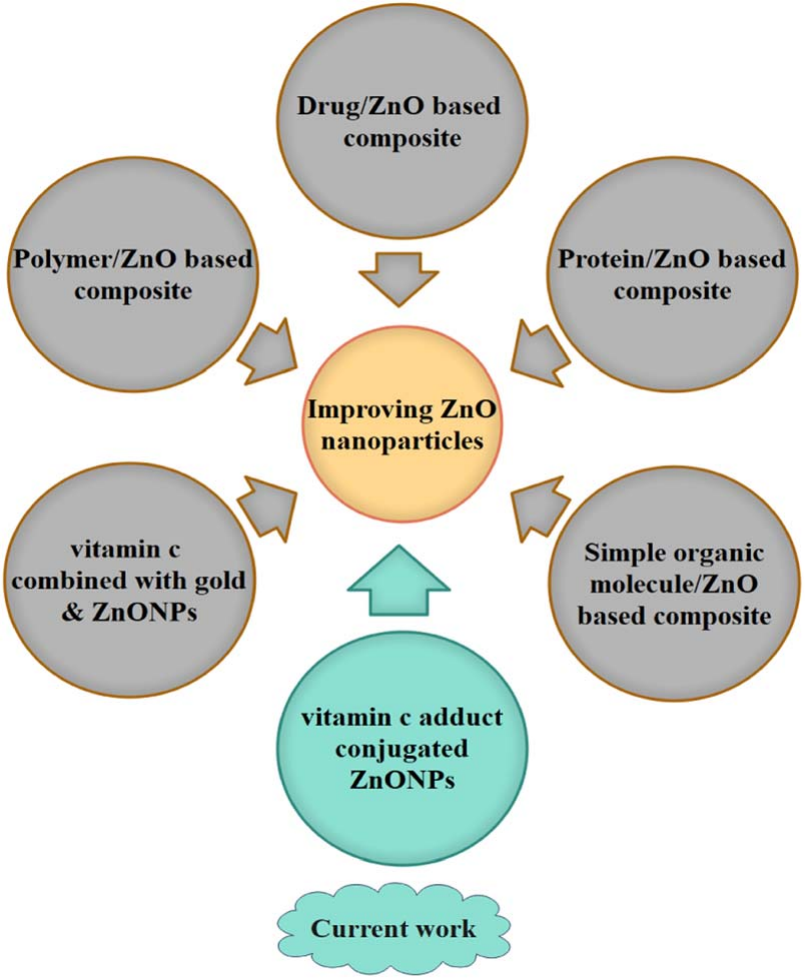
Recent advances in organic compounds conjugated to zinc oxide nanoparticles (NPs) and quantum dots (QDs).

In parallel with the doping process, recent scientific efforts to improve ZnONPs have also revealed the contribution of various organic substrates. Fig. 1 shows the recent advances in electrical, biological, and biomedical applications of conjugated ZnONPs. Intensive efforts have been devoted to polymer/ZnO-based composites.^17^ Many composites of polymers and ZnO have been developed by several researchers, such as polyethylenimine (PEI), polystyrene (PS), poly(3-hexylthiophene) (P3HT), polydimethylsiloxane (PDMS), poly(3,4-ethylenedioxythiophene):poly(styrene sulfonate); (PEDOT:PSS), cellulose nanofibers (CNFs), and polyurethane (PU), coupled with ZnONPs and utilized for various physical and electrical applications, including organic solar cells, UV detectors, sensors, actuators, optoelectronic devices, electrical generator devices, high-strength rubber applications, and protective purposes.^18–23^ The conjugated polymers of palm cellulose nanofibers (CNFs) and polylactic acid (PLA) with ZnONPs have been employed for biomedical and biological applications, including breast cancer detection, antibacterial activity, and food packaging.^22,24^ Furthermore, some polymer@ZnO systems, such as polyaniline/ZnO (PANI/ZnO), have been introduced to expand photocatalytic performance.^25^

Researchers have also developed potent antibiotics by grafting ZnONPs onto medically approved drugs. To date, these have been mostly focused on folic acid (FA) as an organic moiety conjugated with ZnONPs, such as FA@ZnONPs and FA@ZnO QDs. These have been applied in various biological fields, including photodynamic therapy (PDT) using visible-light irradiation, and as antioxidants and antibacterials.^2,3,26–28^ Patra *et al.* functionalized ZnONPs with ciprofloxacin, and they disclosed their potential antibacterial activity.^29,30^ The diagnostic application of a ZnONPs hybrid with streptavidin for HIV detection at an early stage was studied by Avinash Chunduri *et al.*^31^

Further diagnostic properties of a ZnONPs-based protein for breast cancer detection were discovered by Kumar *et al.* Another study of a ZnONPs-based protein was performed by Wu and co-workers to improve the biological activities.^4,32^ Proteins linked to ZnONPs have been utilized for diagnostic, anticancer, and drug-delivery purposes.^33–35^

Recently, only limited research related to ZnONPs/simple organic molecules was performed by Shaibal Mukherjee's group, which developed several simple organic molecules with ZnONPs for designing gas-sensor devices, including a naphthalene-based π-conjugated amine (NBA) mixed with zinc oxide ZnONPs for CO_2_ gas detection at room temperature. Also, organo-di-benzoic acidified zinc oxide (ODBA@ZnO) was fabricated and used as a highly selective detector for CO gas at low temperature. In another attempt, ZnO-loaded tyrosine-functionalized benzene-tricarboxyamide was employed as a sensor for detecting and measuring different concentrations of CO gas.^20,36,37^

A few researchers are focusing on the utilization of vitamin C in combination with nanoparticles. Atanu Chakraborty and Nikhil R. Jana created gold nanoparticles and conjugated them with vitamin C. They claimed that the vitamin C intake at the microscale could protect cells from oxidative stress, while at the millimolar scale, it could actually kill cells by producing peroxides.^38^ In another two studies, ZnONPs and vitamin C together were found to increase the cytotoxicity of food additives more than either vitamin C or ZnONPs alone.^39,40^

In this work, a biodegradable vitamin C adduct was functionalized with benign ZnONPs to synthesize nanocomposite l-ascorbic acid adduct-conjugated ZnONPs. The photocatalytic performance of the ZnONPs was improved (drop in the band gap energy) by conjugation with the ascorbic acid adduct. The as-fabricated ZnONPs@vitamin C adduct was successfully employed under solar irradiation instead of UV light for degradation of the dye Congo red (CR). The chemical composition and identity of the fabricated products, namely ZnONPs, vitamin C adduct, and ZnONPs-loaded vitamin C adduct, were characterized and confirmed by various techniques, including XRD, ^1^H-NMR, ^13^C-NMR, FE-SEM, EDX, elemental mapping, PL, zeta potential, DRS, and FT-IR analysis.

## 2. Experimental section

### 2.1 Chemicals, materials, and methods

All the reagents and compounds were acquired from Sigma-Aldrich and were of analytical quality and used without purification. The precursors of the fabricated zinc oxide nanoparticles were zinc acetate dihydrate (Zn(CH_3_COO)_2_·2H_2_O, ≥98%), sodium hydroxide (NaOH, ≥98%), and acetone ((CH_3_)_2_CO, ≥99.5%). The precursors for the synthesized vitamin C adduct were 4-nitroaniline (O_2_NC_6_H_4_NH_2_, ≥99%), hydrochloric acid (HCl, 37%), sodium nitrite (NaNO_2_, ≥99%), and l-ascorbic acid (C_6_H_8_O_6_, ≥99%). TLC plates were prepared by pasting silica gel on aluminium foil (Merck, grade 60 F254 MS) to monitor the chemical reactions. A UV lamp (254 and 360 nm) was used to display the TLC spots. The IR discs were formed by mixing a minimum amount of the sample with KBr powder and then pressing them under 12 tons of pressure. A PerkinElmer spectrophotometer was used to acquire the FT-IR spectra (Waltham, MA, USA). The ^1^H NMR and ^13^C NMR spectra were obtained using a 500 MHz Bruker Avance DRX spectrometer (Billerica, MA, USA). The structural parameters were analyzed using an X-ray diffractometer (PANanalytical X’PERT-PRO MRD) equipped with a Cu kα (*λ* = 0.15406 nm) source of radiation in the range of 10° to 80° at a scan rate of 2° min^−1^. Using a field-emission scanning electron microscopy system (FE-SEM, Zeiss sigma 300-HV, Oberkochen, Germany), microstructural and chemical analyses of the samples (pure ZnONPS and vitamin C adduct/ZnONPS composite) were carried out with an energy-dispersive X-ray unit (EDX, INCAx-sight, model: 7426, Oxford Instruments, Abingdon, Oxfordshire, UK). Photoluminescence (PL) studies were performed on a FlouTime 300-PicoQuant system (Germany). The absorption spectra were analyzed by UV-vis-diffuse reflectance spectrophotometry (UV-vis-DRS, SCINCO Model S-4100). Zeta potential analysis for assessing the stability of the particles in the colloidal system was carried out using a Horiba Jobin Yvon: Sz 100 system.

### 2.2 Fabrication of zinc oxide nanoparticles

ZnONPs were fabricated by following a previously reported co-precipitation method with a few alterations.^41,42^

Solution (A): Zn(CH_3_CO_2_)_2_·2H_2_O (0.1 M, 100 ml).

Solution (B): NaOH (0.2 M, 100 ml).

Solution (B) was added dropwise into Solution (A) with continuous stirring at 750 rpm for 2 h under a temperature of 70–80 °C. The obtained transparent mixture turned into a white and milky solution. The intermediate (Zn(OH)_2_, white precipitate) was isolated by filtration and purified by deionized water, followed by acetone. Zinc oxide nanoparticles (ZnONPs) were formed by drying the product in a conventional laboratory oven at 75 °C for 8 h and then calcining at 500 °C for 4 h. The fabricated ZnONPs were characterized by FT-IR, XRD, FE-SEM, EDX, UV-vis-DRS, PL, and zeta potential.

### 2.3 Synthesis of l-ascorbic acid adduct (3): 15-hydroxy-12-oxotetrahydrofuran-14-yl11-(1-(4-nitrophenyl)hydrazineyl)-10-oxoacetate

The synthesis of the of l-ascorbic acid adduct (3) was carried out in accordance with the procedure described by Duncan L. Browne, Ian R. Baxendale, and Steven V. Ley.^43^ First, 4-N\nitroaniline (6.90 g, 50 mmol, 1 equiv.), 30 ml distilled water, and 30 ml concentrated hydrochloric acid were added to a reaction vessel and agitated until the reactant dissolved completely. The solution was cooled to 0–5 °C, and sodium nitrite solution NaNO_2_ (6.90 g, 100 mmol, 2 equiv.) was added dropwise using a burette. The mixture was stirred for 1 h, and a clear yellow solution of diazonium salt was obtained by filtration. To the resulting solution, l-ascorbic acid (22 g, 125 mmol, 2.5 equiv., 100 ml H_2_O) was added dropwise (20 min) by burette. The obtained solution was stirred for a further 1 h and then filtered. The crude product (16.55 g, 94%) was purified by recrystallization with water to obtain the pure vitamin C adduct (15.85 g, 90%, yellow precipitate). The obtained product (3) was characterized by FT-IR, XRD, ^1^H-NMR, ^13^C-NMR, FE-SEM, EDX, and UV-vis-DRS. ^1^H-NMR (500 MHz, dmso) *δ* 11.31 (s, 1H), 9.31 (s, 1H), 8.10 (d, *J* = 8.9 Hz, 2H), 6.82 (d, *J* = 8.8 Hz, 2H), 6.19 (s, 1H), 5.71 (d, *J* = 7.9 Hz, 1H), 4.71 (q, *J* = 8.0 Hz, 1H), 4.52 (dd, *J* = 8.18, 7.74 Hz, 1H), 4.08 (dd, *J* = 8.30, 8.33 Hz, 1H). ^13^C-NMR (126 MHz, dmso) *δ* 170.64, 158.87, 156.20, 154.09, 139.08, 126.35, 111.42, 76.54, 70.11, 69.81.

### 2.4 Synthesis of vitamin C adduct-conjugated ZnONPs (4)

First, 0.1 g of the previously prepared adduct (3) was dissolved in 5 ml acetonitrile in a 25 ml cylindrical vial with constant stirring. The solution was diluted by adding 5 ml of distilled water, followed by adding 1 g of the previously prepared ZnONPs. The vial was sealed, and the mixture was stirred for 6 h at ambient temperature. The reaction mixture was then centrifuged for 7 min at 4500 rpm, and then the filtrate was removed by decantation, and the precipitate was recovered by washing (3 × 10 ml, cold distilled water). The precipitate was dried in an oven at 75 °C overnight. The composite (3) was characterized using FT-IR, FE-SEM, XRD, EDX, PL, zeta potential, DRS, and UV-vis spectroscopy.

### 2.5 Photocatalytic activity of ZnONPs and ZnONPs@vitamin C adduct for the degradation of Congo red organic dye

Two different experiments under solar light LED bulbs (15 W) were conducted to evaluate the photocatalytic activity of both ZnONPs and ZnONPs@vitamin C adduct toward the organic substrate Congo red (CR).

### 2.6 Experimental procedure

First, 100 ml of Congo red solution (50 ppm) and 50 mg of the catalysts (ZnO/ZnONPs@vitamin C adduct) were added together, and the resultant solution had a pH range of 7.0–7.8. The suspension solution was left in the dark with continuous stirring for 30 min to attain sorption–desorption equilibrium. The suspended solution was irradiated with solar light throughout the study while magnetically stirring at 500 rpm. The absorption of the samples was measured at *λ*_ma*x*_ = 497 nm with a UV-vis spectrophotometer every 15 min for a total time of 180 min.

## 3. Results and discussion

### 3.1 Chemical synthesis: synthesis of the ZnO@vitamin C adduct (4)

**Scheme 1:**
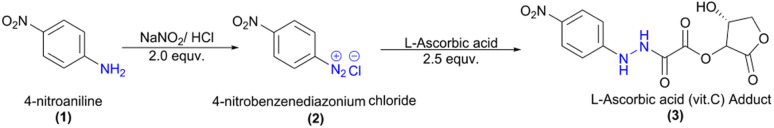
Synthesis of the l-ascorbic acid (vitamin C) adduct.

The l-ascorbic acid adduct (3) was synthesized *via* several steps, as described in Scheme 1. Initially, 4-nitroaniline (1) was protonated by an excess of concentrated hydrochloric acid (HCl), and then the unreacted materials were removed by filtration. The reaction mixture was diazotized by sodium nitrite (NaNO_2_) to form a diazonium salt (2). Compound (2) was reacted with excess (2.5 equiv.) of l-ascorbic acid to produce the adduct (3). The chemical composition and morphology of the purified compound (3) were confirmed by FT-IR, ^1^H-NMR, ^13^C-NMR, PL, FE-SEM, EDX, elemental mapping, DRS, and X-ray diffraction.

**Scheme 2:**
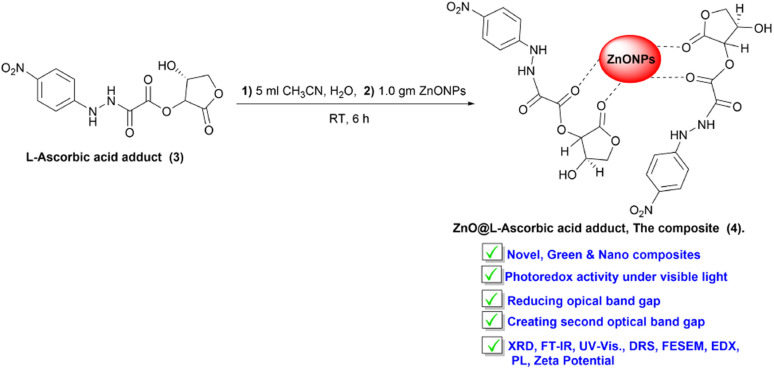
Synthesis of the vitamin C adduct-conjugated ZnONPs.

ZnO nanopowder was added to the purified l-ascorbic acid adduct solution to produce the ZnONPs-conjugated l-ascorbic acid adduct (4), as outlined in Scheme 2. The structural and chemical information of the composite (4) was emphasized and characterized by various techniques, including FT-IR, FE-SEM, XRD, EDX, elemental mapping, PL, zeta potential, DRS, and UV-visible spectroscopy.

### 3.2 Characterizations

#### 3.2.1 FT-IR study

The absorption of infrared radiation raises the vibrational energy of molecules from lower levels to higher ones.^44^ IR signals are generated from these vibrational transitions and can be used to identify the organic functional groups.^45^

**Fig. 2:**
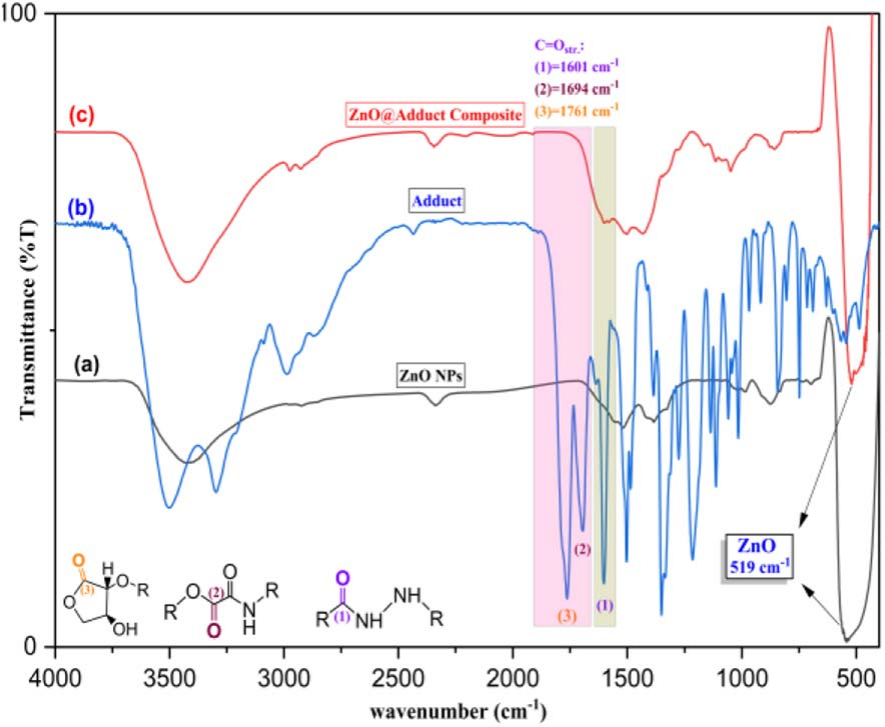
FT-IR spectra of (a) ZnONPs, (b) l-ascorbic acid adduct, and (c) l-ascorbic acid adduct-conjugated ZnONPs, respectively.

Fig. 2a depicts the FT-IR spectrum of ZnONPs pretreated at 500 °C for 4 h. The characteristic peak at 519 cm^−1^ was for Zn–O vibrational stretching. This was accompanied by an IR signal at 3424 cm^−1^ for the vibrational stretching of the hydroxyl group (O–H_str._) for ZnONPs, further confirming for the formation of nanoparticles of ZnO.^46^ The important organic functional groups in l-ascorbic adduct (3) are shown in Fig. 2b. The functional group region of the IR spectrum displayed a sharp and intense vibration at a high frequency (3499 cm^−1^) for O–H_str._ and amide N–H_str._ at 3299 and 3210 cm^−1^, respectively, because the O–H group contributed less to forming hydrogen bonds than the N–H group in the adduct. Both the sp^2^ and sp^3^ carbons involved in the C–H bond showed vibrational stretching signals at 3088 and 2990 cm^−1^, respectively.

The appearance of C=O stretching was the most valuable signal in the IR spectrum and confirmed the formation of the l-ascorbic acid adduct. The stretching signals at 1764, 1694, and 1601 cm^−1^ were due to the existence of the three carbonyl groups in the adduct (3).^43^ The strength order of the esteric carbonyl stretching in compound (3) is shown in Fig. 2b. Both C_(3)_=O_str._ and C_(2)_=O_str._ had relatively higher wave numbers due to the inductive effect of the electronegative oxygen atoms, appearing at 1764 and 1694 cm^−1^, respectively, while the amide C_(1)_=O_str._ appeared at 1601 cm^−1^ because of the dominant mesomeric effect.^47^ Further confirmation of the adduct molecular structure could be obtained from the IR spectrum fingerprint region. The C–O and C–N stretches appeared in the range of 1351 and 1214 cm^−1^, respectively. Fig. 2c confirmed the fabrication of composite (4), l-ascorbic acid adduct-conjugated ZnONPs. The disappearance of IR bands was very helpful and provided remarkable information about the fabricated compound (4). Astonishingly, the two signals attributed to esteric C=O_str._ at 1761 and 1694 cm^−1^ in the l-ascorbic acid adduct-conjugated ZnONPs disappeared, proving the linkage between the vitamin C adduct and ZnONPs was through the two esteric carbonyl groups (see Scheme 2). Similar outcomes were reported by El-Borady *et al.* and Rana *et al.*^3,48^

#### 3.2.2 XRD study

**Fig. 3:**
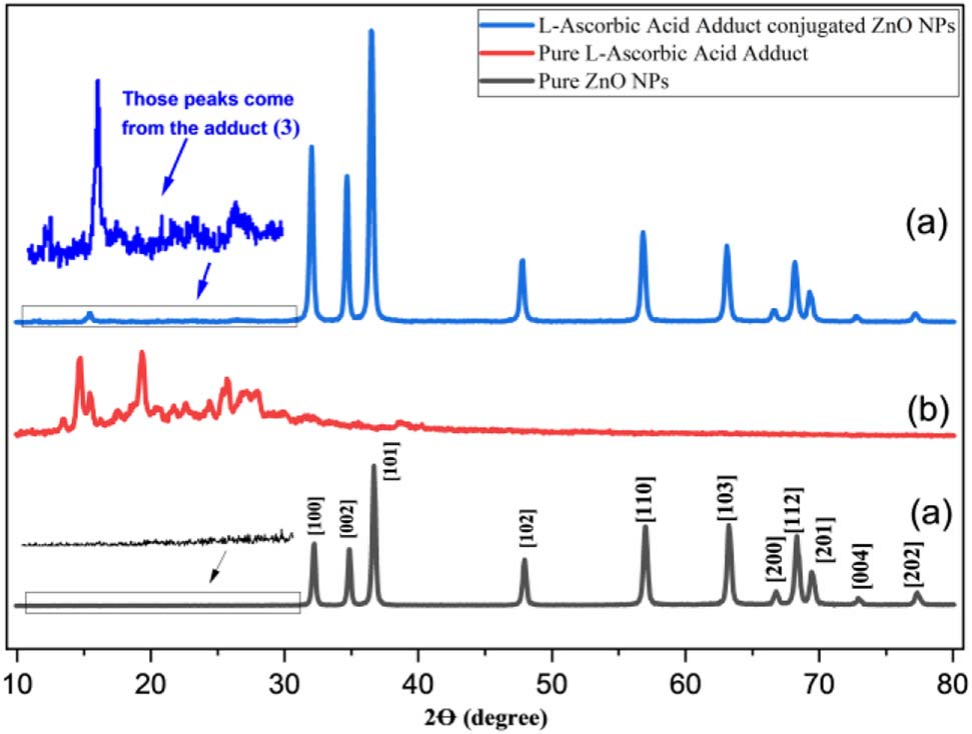
XRD spectra of (a) ZnONPs (black), (b) l-ascorbic acid adduct (red), and (c) l-ascorbic acid adduct-conjugated ZnONPs (blue), respectively.

Fig. 3 presents the XRD diffraction patterns of the pure ZnONPs (black), prepared l-ascorbic acid adduct (red), and l-ascorbic acid adduct-conjugated ZnONPs (composite) (blue). The diffraction patterns of the synthesized ZnONPs exhibited distinctive diffraction peaks at (2*θ*) 32.2°, 34.8°, 36.6°, 47.9°, 56.9°, 63.2°, 66.7°, 68.3°, 69.4°, 72.9°, and 77.3°, which could be indexed to the typical (100), (002), (101), (102), (110), (103), (200), (112), (201), (004) and (202) crystal plane of pure ZnO, respectively (Fig. 3a).

It could be noticed that the reflection planes of the fabricated ZnO nanopowder were exactly comparable to those of (JCPDS card: 36-1451). The strong and distinct peaks indicated that the acquired ZnO nanoparticles were well crystallized, while the absence of distinctive peaks resulting from reactants, other foreign particles, or ZnO in other phases indicated the excellent purity of the ZnO generated by this precipitation process. Scherrer's equation was used to provide an estimate of the average crystalline size (*D*) of the ZnO nanoparticles: *D* = *k λ*/*β* cos *θ*, where *k* is a constant equal to 0.90, *λ* is the wavelength of X-rays equal to 0.15406 nm, *β* is the full width at half maximum, and *θ* is half the diffraction angle. According to the calculation, the average particle size (*D*) was around 23 nm. In Fig. 3b, broad diffraction peaks could be observed in the range of 2*θ* = 15–30° for the synthesized l-ascorbic acid adduct, indicating the amorphous behaviour of organic compounds. It is worth mentioning that a similar appearance of organic amorphous signals was observed by Mandal *et al.*^20^ and Sun *et al.*^49^ The appearance of new signals (15° to 27°) for the organic part in compound (4) (see Fig. 3c) confirmed the formation of pure composites (4) with the remaining showing the wurtzite crystalline structure of ZnONPs. The obvious signal of the organic moiety at 2*θ* equal to 15.4° indicated the linkage between ZnONPs and l-ascorbic acid without any impurities. Several researchers have fabricated other ZnONPs@organic composites, and have reported the appearance of only a few strong signals among all the signals that belong to organic compounds, most likely due to the existence of intense peaks of the ZnONPs.^22,50–52^ The average crystallized size (*D*) of the composite was calculated according to Scherrer's equation and was determined as 23.5 nm.

#### 3.2.3 ^1^H-NMR and ^13^C-NMR studies

##### 3.2.3.1 ^1^H-NMR study

**Fig. 4:**
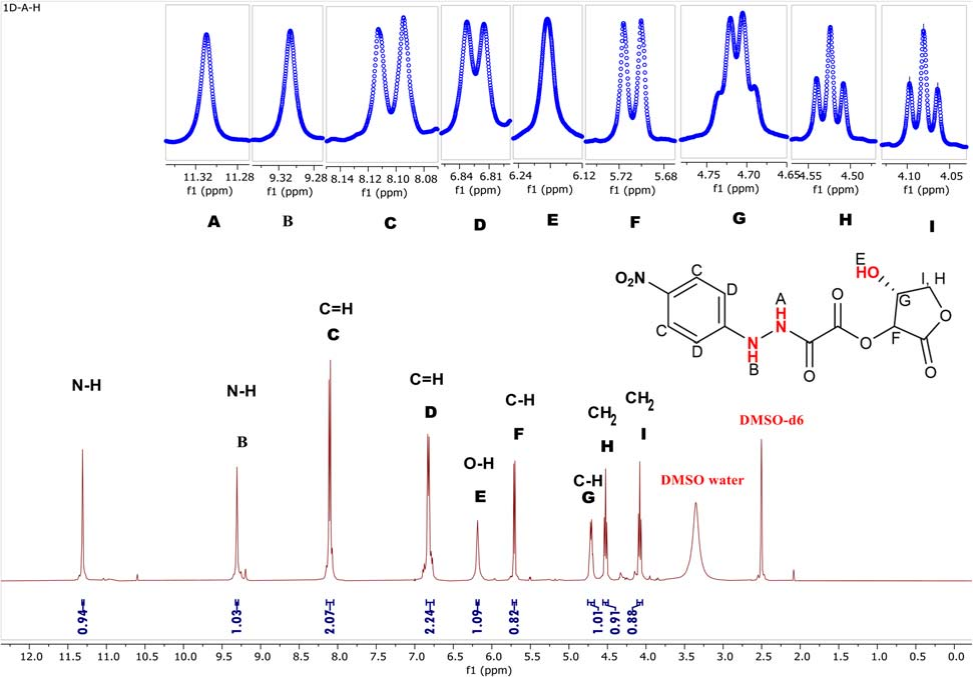
^1^H-NMR spectrum of (15-hydroxy-12-oxotetrahydrofuran-14-yl11-(1-(4-nitrophenyl)hydrazineyl)-10-oxoacetate) in DMSO-d_6_ solvent.

The adduct (3) hydrogens were determined and characterized by ^1^H-NMR spectroscopy. The ^1^H-NMR spectrum (Fig. 4) of the vitamin C adduct was obtained by dissolving the sample in DMSO-d_6_. The two exchangeable hydrogens (N–H and O–H) in compound (3) were seen as three signals in the range *δ* = 11.31–6.19 ppm. The two hydrogen atoms of N–H resonated at higher frequencies (*δ* = 11.31 and 9.31 ppm) as two singlets. These may be related to the presence of hydrogen bonding; this understanding is compatible with the IR information for adduct (3) (see Fig. 2b).

The (O–H) proton resonated as a singlet (*δ* = 6.19 ppm).^53^ The four aromatic protons appeared as two signals; with two chemical equivalency protons resonating as a doublet at *δ* = 8.11 ppm, while the remaining two were detected at *δ* = 6.81 ppm. The rest of the protons in the range of 5.75 to 4.00 ppm were related to the lactone moiety. The two diastereotopic protons (CH_2_) appeared as two different fake triplet signals at *δ* = 4.08 and 4.52 ppm, respectively.^47^ Further spectral investigations of the symmetrical signals showed the presence of two hidden doublets of doublet signals. The first diastereotopic proton resonated at 4.52 ppm and had real ^1^H-coupling (dd, *J* = 8.18, 7.74 Hz), while the other diastereotopic proton appeared at 4.08 ppm and illustrates barely any spin coupling (dd, *J* = 8.30, 8.33 Hz, 1H). A similar observation was recorded by Yaseen Ahmed and Omer Rashid.^47^ The protons of two asymmetric carbons resonated at 5.71 (d, *J* = 7.9 Hz, 1H) and 4.71 (q, *J* = 8.5 Hz, 1H), respectively. The ^1^H-NMR signals of the solvent DMSO-d_6_ appeared as two signals at *δ* = 3.35 ppm (DMSO–water) and a characteristic proton signal at *δ* = 2.49 ppm.^54^

##### 3.2.3.2 ^13^C-NMR study

**Fig. 5:**
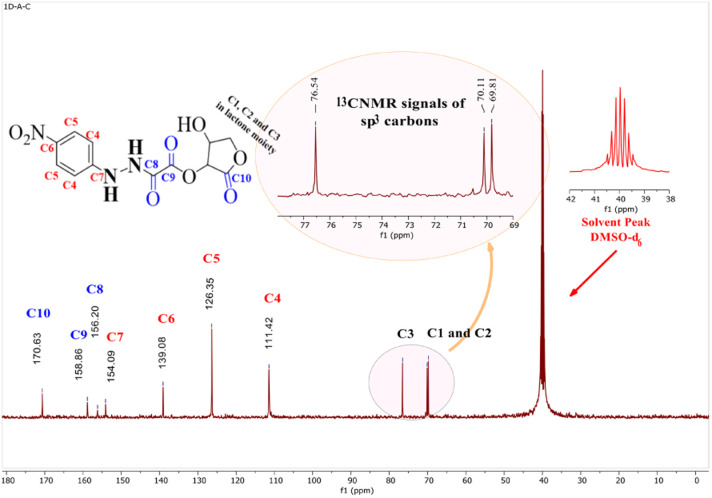
^13^C-NMR spectrum of (15-hydroxy-12-oxotetrahydrofuran-14-yl11-(1-(4-nitrophenyl)hydrazineyl)-10-oxoacetate) in DMSO-d6 solvent.

^13^C-NMR spectroscopy was used to validate the carbon skeleton in compound (3), as depicted in Fig. 5. The carbons in the aromatic ring possessed four signals: (1) two carbons (C_5_) in the ortho positions of the nitro group, which were more deshielded and appeared at *δ* = 126.35 ppm, (2) another two aromatic carbons (C_4_), which were detected at *δ* = 111.42 ppm (C_7_), (3) the quaternary aromatic carbon adjacent to the hydrazine group, which were detected at *δ* = 154.09 ppm, and (C_6_), the quaternary carbon close to the nitro group, which was detected at *δ* = 139.08 ppm. Three signals of the carbon in the carbonyl groups (C=O) were also visible.

Lactonic carbonyl (C_10_=O) had the most deshielded signal (*δ* = 170.63 ppm) in the spectrum, while esteric carbonyl (C_9_=O) resonated at *δ* = 158.86 ppm, and the amidic carbonyl (C_8_=O) appeared at *δ* = 154.09 ppm.

These results were consistent with the ^13^C-NMR analysis for the compounds synthesized and analyzed by Browne *et al.*^43^ The three signals at *δ* = 69.81, 70.11, and 76.54 ppm, respectively, validated the remaining carbons (C_1_, C_2_, and C_3_).

#### 3.2.4 FE-SEM, elemental mapping, and EDX studies

**Fig. 6:**
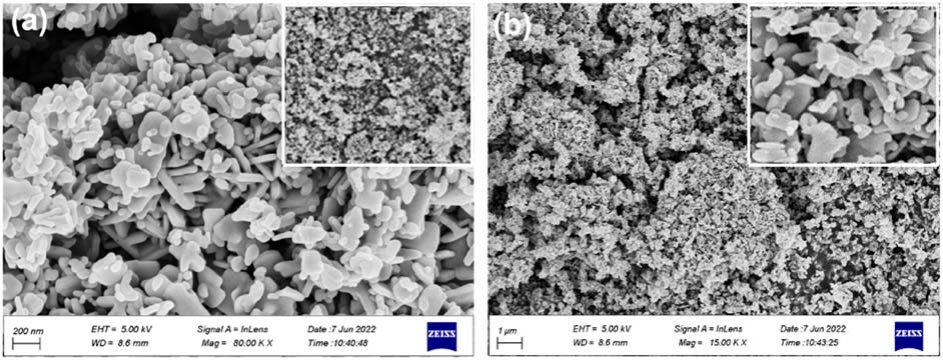
FE-SEM images (a & b) of the fabricated ZnONPs at different magnifications.

**Fig. 7:**
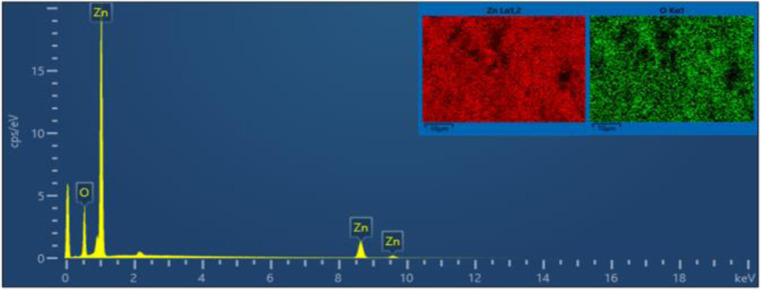
EDX spectrum and elemental analysis of the fabricated ZnONPs.

**Fig. 8:**
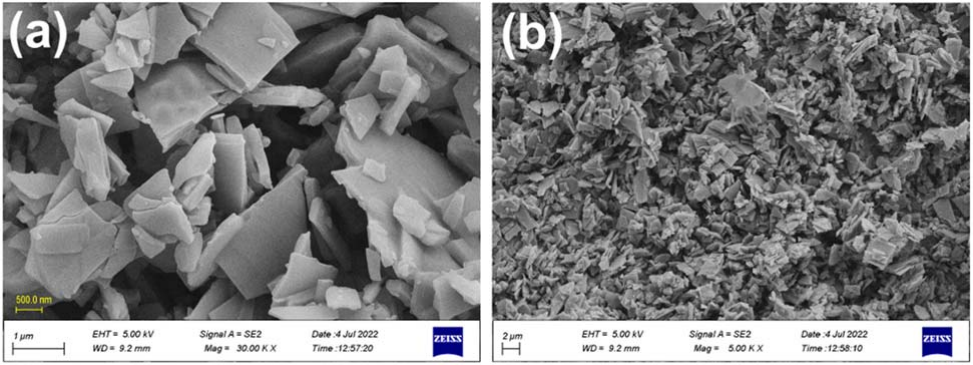
FE-SEM images (a & b) for the synthesized l-ascorbic acid adduct (3) at different magnifications.

**Fig. 9:**
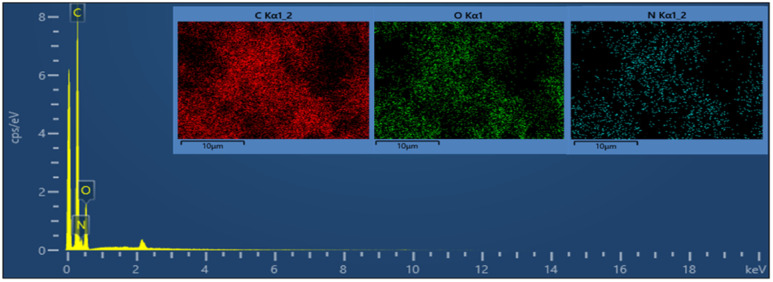
EDX spectrum and elemental analysis of the synthesized l-ascorbic acid adduct (3).

**Fig. 10:**
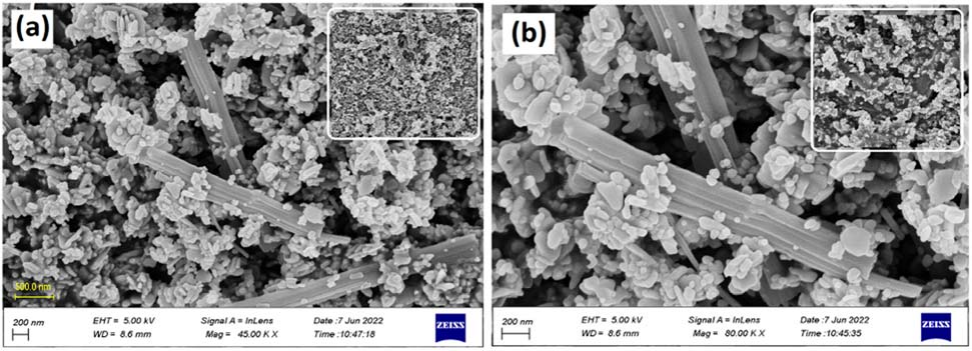
FE-SEM images of the synthesized composite at different magnifications.

**Fig. 11:**
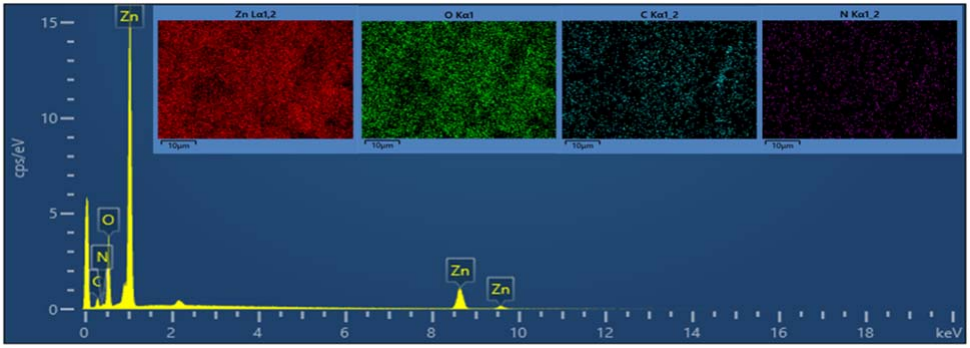
EDX spectrum and elemental analysis of the synthesized composite.

Field-emission scanning electron microscopy (FE-SEM) is a powerful technique for investigating the surface morphology of NPs. The FE-SEM images of the fabricated ZnONPs, l-ascorbic acid adduct (3), and nanocomposite (4) are illustrated in Fig. 6–11.

FE-SEM morphology analysis of the fabricated ZnONPs was performed and the results are demonstrated in different magnifications in Fig. 6a and b. Accordingly, the particle size ranged from 8 to 50 nm and exhibited a plate-like structure arranged in a regular manner; however, few particle agglomerations could be observed. The energy-dispersive X-ray (EDX) spectrum of ZnONPs is depicted in Fig. 7 and the results listed in Table 1. The spectrum and mapping images showed the successful formation of ZnONPs without any impurities.

**Table 1:** Elemental analysis results of the fabricated pure ZnONPs

Mapping results summary				
Element	Line type	Weight%	Weight% sigma	Atomic%
O	K series	22.88	0.23	54.80
Zn	K series	77.12	0.23	45.20
Total		**100.00**		**100.00**

Fig. 8a and b show the FE-SEM photos of the synthesized adduct (3), showing the particles had random shapes consisting mostly of disorganized square plates with a mean diameter of 50 to 200 nm. The EDX spectrum of compound (3) is displayed in Fig. 9 and the results are listed in Table 2. Both the EDX spectrum and mapping scans revealed the successful synthesis of the adduct (3). The presence of only the elements carbon (weight% = 67.89%), nitrogen (weight% = 1.13%), and oxygen (weight% = 30.98%) confirmed that the pure compound (3) was obtained. The FE-SEM, elemental mapping, and EDX spectra of the fabricated composite (4) are presented in Fig. 10, 11 and Table 3. Predictably, neither the form nor the size of the ZnO nanostructures was appreciably altered by the surface functionalization and loading procedure.

**Table 2:** Elemental analysis of the pure l-ascorbic acid adduct (3)

Mapping results summary				
Element	Line type	Weight%	Weight% sigma	Atomic%
C	K series	67.89	1.73	73.70
O	K series	30.98	0.88	25.25
N	K series	1.13	2.43	1.06
Total		**100.00**		**100.00**

**Table 3:** Elemental analysis of ZnONPs@vitamin C adduct (4)

Mapping results summary				
Element	Line type	Weight%	Weight% sigma	Atomic%
O	K series	24.32	0.29	41.84
Zn	L series	60.83	0.46	25.61
C	K series	10.31	0.44	23.64
N	K series	4.53	0.42	8.91
Total		**100.00**		**100.00**

Whereas a significant change in the shape, size, and morphology of the vitamin C adduct could clearly be seen, as evidenced by comparing the FE-SEM images of the ZnONPs, adduct, and those of the composite.

This remarkable structural alteration may be due to the breakdown of the hosted adduct (3) during the fabrication of compound (4) and then assembling as a template rod-like structure within composite (4). It is worth noting that the FE-SEM images in Fig. 10a and b show two phases: the plate-like structure of ZnO and the rod-like structure of ascorbic acid adduct (3), within the composite (4). The existence of the elements Zn (60.83), O (24.32), C (10.31), and N (4.53) and their percentage weights showed the successful preparation of the ZnONPs@l-ascorbic acid adduct without any impurities. These results match with the other spectroscopic data from the FT-IR and XRD analyses.

#### 3.2.5 UV-vis-DRS study

**Fig. 12:**
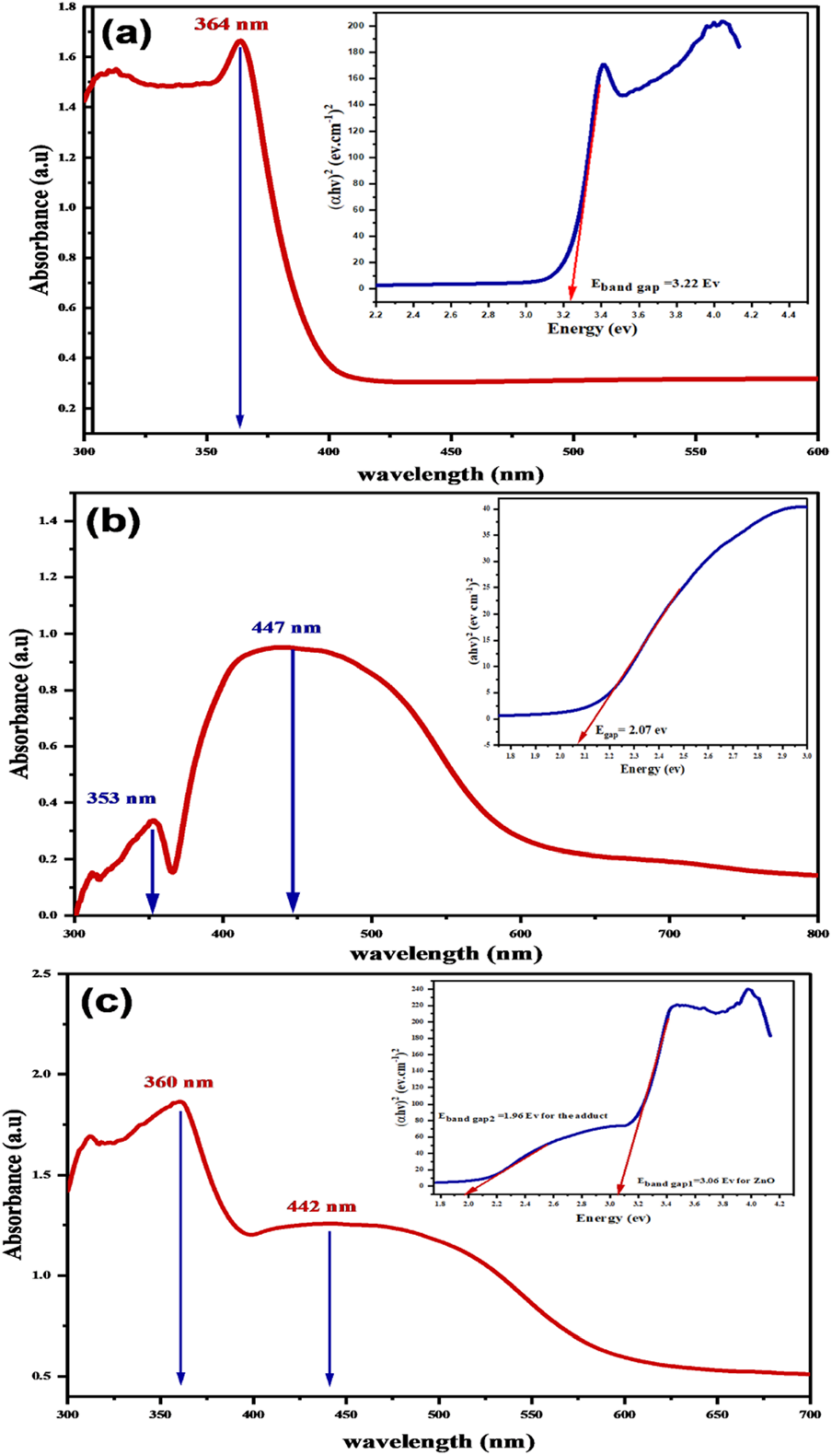
UV-visible-DRS Spectrum and Tauc plots of the (a) ZnONPs, (b) l-ascorbic acid adduct, and (c) l-ascorbic acid adduct/ZnO composite.

UV-visible-DRS was used as an invaluable spectroscopic tool to investigate the physical properties of the absorption spectra of the ZnO nanoparticles,^55^l-ascorbic acid adduct, and the ZnONPs-conjugated vitamin C adduct, see Fig. 12a–c. Fig. 12a shows there was an absorption band at 364 nm owing to the existence of ZnONPs in the UV-vis absorption spectrum. The band gap energy (*E*_band gap_) of ZnONPs (3.22 eV) was thus estimated as the position where the Tauc plot tangent began. Fig. 12b illustrates the UV-vis-DRS results of the adduct (3). The diffuse reflectance spectra exhibited two absorption bands: the UVA band at 353 nm and the visible absorption band at 447 nm (yellowish orange). Simply, the adduct band energy (*E*_band gap_) was determined by Tauc plot and was equal to 2.07 eV. The UV-vis absorption spectrum of the composite (4) is shown in Fig. 12c. The first absorption band at 360 nm indicated the photonic activity of ZnONPs in the UV region,^56^ while the interesting visible absorption band at 442 nm was related to the optical activity of the adduct within the composite (4).

The computed band gap energies (*E*_band gap_) of the ZnO and adduct were 3.06 and 1.90 eV, respectively. Notably for composite (4), the band gap energy declined from 3.22 eV (pure ZnONPs) to 3.06 eV. Additionally, the insertion of the organic moiety (3) inside the nanocomposite (4) formed a new band gap energy (1.96 eV), which was a little bit smaller than that of the prepared adduct (2.07 eV). The existence of two absorption peaks and two band gap energies in the composite spectrum indicated that ZnO and the adduct had been successfully coupled. This outcome would suggest that the novel nanocomposite would be photocatalytically active under visible-light irradiation.

#### 3.2.6 Photoluminescence (PL) study

**Fig. 13:**
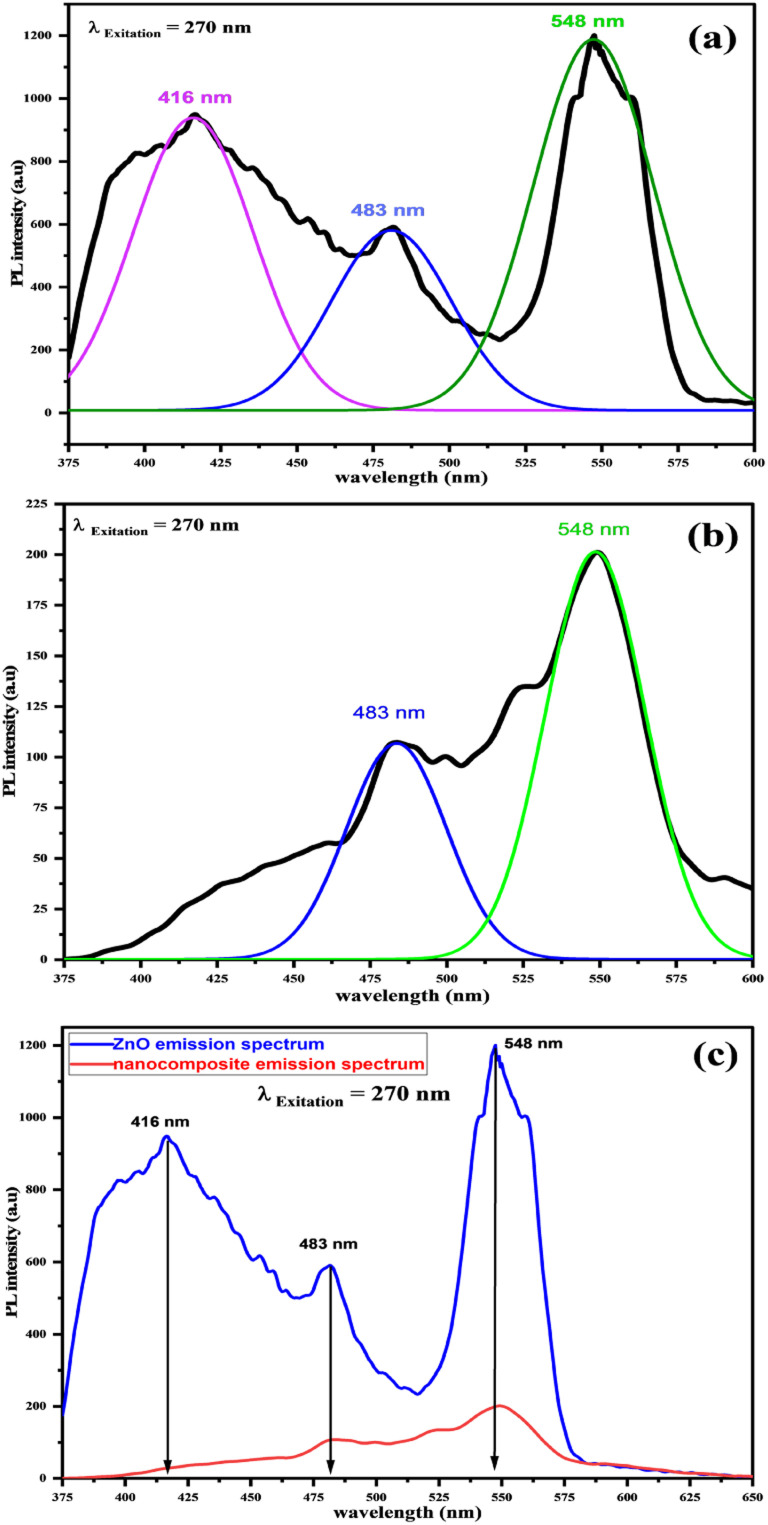
PL spectra of (a) pure ZnONPs and (b) ZnO@l-ascorbic acid adduct. (c) Stacked emission spectra of ZnO (blue) and the composite (red).

Photoluminescence (PL) is a fascinating protocol since it provides exceptional information on the purity and quality of materials.^57^ The photoluminescence spectra of ZnONPs and ZnO-conjugated l-ascorbic acid adduct are shown in Fig. 13a–c. The PL investigation for free ZnONPs was performed at the wavelength of 270 nm (see Fig. 13a). The PL spectrum of ZnONPs showed an emission peak at 417 nm (violet), which was the typical PL emission peak of ZnONPs.^58^ The system of free excitons recombination generates this peak through an exciton–exciton collision mechanism or by inherent defects between zinc and oxygen (Zn–O interstitials).^59,60^ Another peak arose at 480 nm (blue), resulting from the radiated recombination between the photogenerated e^−^/h^+^ populating the oxygen vacancy, while a third emission peak appeared at 548 nm. The green fluorescence seen was caused by oxygen defect sites on the ZnO nanoparticles.^61^

When ZnONPs were conjugated with an organic moiety (3), the PL spectrum changed, while the emission was recorded at the same excitation wavelength (270 nm) as pure ZnO (see Fig. 13b). The excitation at 270 nm produced two peaks at 483 (blue) and 548 nm (green). When the intensity of the composite peaks was compared to the intensity of the ZnO emission peaks (see Fig. 13c), a decrease in peak intensity was noted; even so, it is understood that the decrease in the PL intensity causes a decrease in the rate recombination of photo-induced electron–hole pairs, resulting in a stronger photocatalytic performance.^62^ As a result, the ZnONPs@vitamin C adduct outperformed free ZnONPs in terms of catalytic properties. The total PL outcomes demonstrated the existence of the luminescence property of ZnONPs even after connecting to the organic moiety (3), and also an expansion of the luminescence range. The physicochemical interaction between ZnONPs with the l-ascorbic acid adduct allowed for a much more effective charge transportation for the photogenerated electron–hole (e^−^/h^+^) pairs. As a result, this could lead to an increase in the catalytic performance when exposed to solar radiation. It was seen that the strength of the emission peaks changed due to the interaction with the adduct (3), indicating the growth in surface defects. As a consequence, the optical characteristics of the produced nanocomposite were altered.

#### 3.2.7 Zeta-potential study

**Fig. 14:**
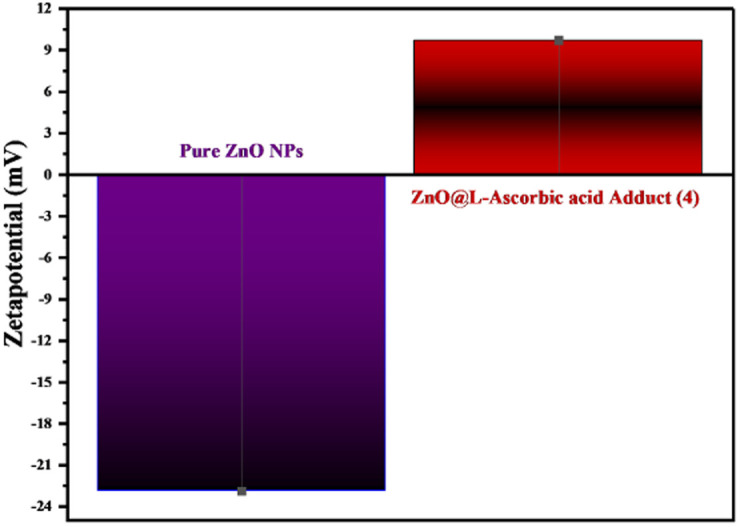
Representation of the zeta-potential values for both ZnONPs and the synthesized composite, showing the reversed signs from minus to plus.

The relevance of the zeta potential lies in the correlation between its value and the short- and long-term stability of emulsions. The lifetime stability of NPs is strongly related to the surface charge distribution. In order to determine the zeta potential in the current investigation, dispersed ZnO nanoparticle and ZnO@l-ascorbic acid adduct solutions were separately applied to the electrode along with setting the experimental parameters, such as conductivity (0.065 and 0.111 mS cm^−1^) and electrode voltage (3.9 and 3.4 V), respectively, while the other conditions were kept the same for both solutions, such as pH range (7.0–7.5), temperature (25.10 °C), and dispersion medium (DI) viscosity (0.893 mPa s). The experimental data indicated that the ZnONPs were stable, with a mean zeta potential of −22.9 mV for the ZnONPs and +9.7 mV for the composite (4) (Fig. 14). The surface of the nanoparticles was seen to be negatively charged and spread throughout the liquid. The zeta-potential value for the ZnONPs validated the repulsion between the particles and demonstrated their reasonable stability,^63^ while the zeta-potential value for the composite was positive (+9.7 mV) and lower than that of pure ZnONPs. The FE-SEM photos of the composite (4) (Fig. 10) verified the presence of agglomerations when ZnO was loaded on the surface of the adduct (3). As noted, the zeta-potential value was highly dependent on the pH, temperature, and sample preparation medium.^64–66^ Therefore, further study is required to enhance the surface charge and stability of the fabricated composite (4). As mentioned above, the zeta potential of ZnONPs was −22.9 mV, but it was +9.7 mV upon loading with the adduct and the formation of the composite (4) due to the positive charge of the adduct (see Table 4),^67^ and this flip from a negative to positive surface charge suggested that ZnO was effectively loaded onto the surface of the l-ascorbic acid adduct. We could thus confirm the successful fabrication of composite (4) by conjugation between ZnONPs and the adduct (3). This result exactly fits with the information obtained from the FT-IR, XRD, and FE-SEM studies.

**Table 4:** Zeta potential values and electrophoretic mobility for pure ZnONPs and the composite

Compounds	Zeta potential (mean) (mV)	Electrophoretic mobility (mean) (cm^2^ V^−1^ s^−1^)
Pure ZnONPs	−22.9	−0.000177
ZnO@l-ascorbic acid adduct	+9.7	+0.000075

### 3.3 Photocatalytic degradation study

Electrons in the valence band (VB) can be excited to the conduction band (CB) by photons with energy equal to or larger than the band gap, resulting in the formation of electron–hole pairs (e^−^/h^+^) as expressed by the following equation:MO + photons → e^−^ + h^+^

**Fig. 15:**
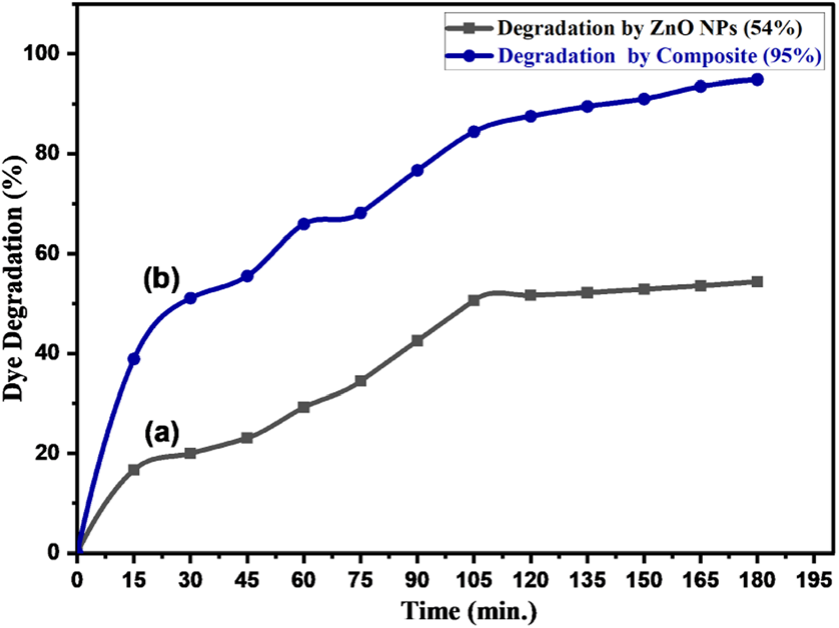
Photocatalytic degradation of Congo red (50 ppm) by (a) pure ZnONPs (0.05 g) and (b) ZnONPs@vitamin C adduct (0.05 g) under solar irradiation, pH 7–8.

The photocatalytic activities of ZnONPs and ZnONPs-conjugated l-ascorbic acid adduct (4) were evaluated by determining the remaining concentration of the CR dye in aqueous solution, whereby the degradation percentage was calculated using the following equation:Degradation (%) = (*C*_o_ − *C*/*C*_o_) × 100where *C*_o_ is the Congo red initial concentration (50 ppm) without the catalyst, and *C* is the dye concentration with the catalyst irradiated by LED light bulbs (15 W) equivalent to solar light at regular intervals. Fig. 15 shows there was a shift in concentration during the photodegradation of CR in the presence of both the synthesized ZnONPs and ZnONPs conjugated to the l-ascorbic acid adduct (4). The absorbance of the CR dye solution progressively dropped as the irradiation time increased, as predicted by the degradation principle. Approximately 30% of the CR dye was degraded within 60 min of treatment with the pure ZnONPs, while 66% of the dye was degraded after treatment with compound (4). The degradation reached 54% and 95% after 180 min for both the ZnONPs and ZnO NPs@vitamin C adduct, respectively.

**Fig. 16:**
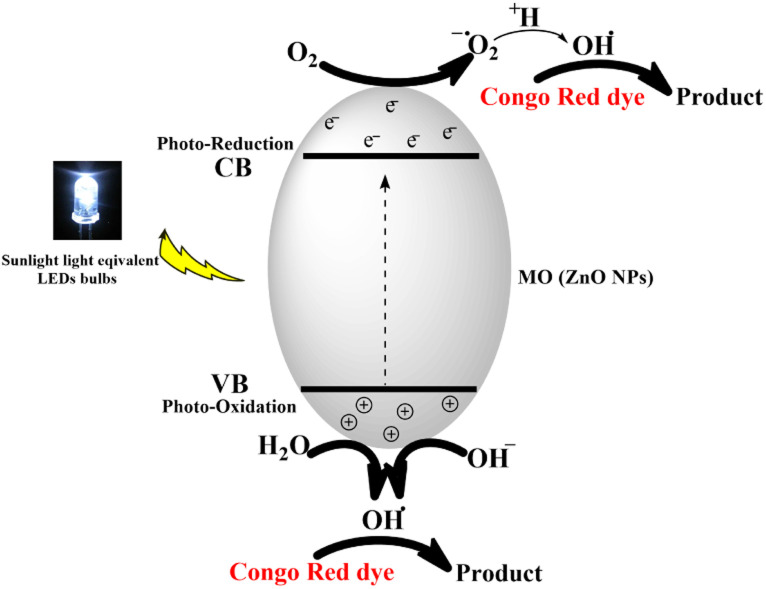
Schematic diagram of the proposed photocatalytic degradation process of Congo red dye.

The photocatalytic degradation mechanism of CR dye by ZnONPs under solar radiation was also reported by Rania E. Adam *et al.*^41^ The produced species, hydroxyl radicals (OH˙), superoxide radical anions (O_2_˙^−^), and hydroperoxyl radicals (HO_2_˙) have been reported to be responsible for the degradation process.^41,68^ The proposed photocatalytic process is shown in Fig. 16.

#### 3.3.1 Role of the vitamin C adduct in the enhancement of the photocatalytic activity of ZnONPs

The improvement in the photocatalytic degradation of CR dye is derived mostly from the enhanced adsorption of CR on the zinc oxide surface through it incorporating the dye into its cavities and adsorbing it onto the surface of the ZnONPs. It could also serve as a bridge or channel for the dye to reach the surface of ZnONPs and gather in higher concentrations, thereby facilitating the degradation of the dye in the presence of active radicals generated during the photocatalytic degradation processes.^69^ Compared to ZnONPs, the photoluminescence intensity of the ZnONPs@l-ascorbic acid adduct decreased, as confirmed by the PL spectrophotometer (Fig. 13c), indicating that the electron/hole recombination was significantly suppressed in the ZnONPs@l-ascorbic acid adduct nanocomposite systems. As electron–hole recombination was diminished, the photocatalytic performance of the ZnONPs was boosted, generating even more active radicals.^70,71^ Consequently, CR degradation was expedited, leading to the creation of mineralized products.^69,72^ In addition, as seen in the UV-vis-DRS results (Fig. 12), the band gap energy of ZnONPs dropped from 3.2 to 3.06 eV after coupling with the vitamin C adduct, and a second band gap (1.96 eV) was formed, which was attributed to the vitamin C adduct within the fabricated composite (4). Finally, under solar and visible-light irradiation, the photocatalytic performance of the ZnONPs coupled with the l-ascorbic acid adduct was significantly raised compared to the ZnONPs alone.

#### 3.3.2 Adsorption–desorption equilibrium in the dark

**Fig. 17:**
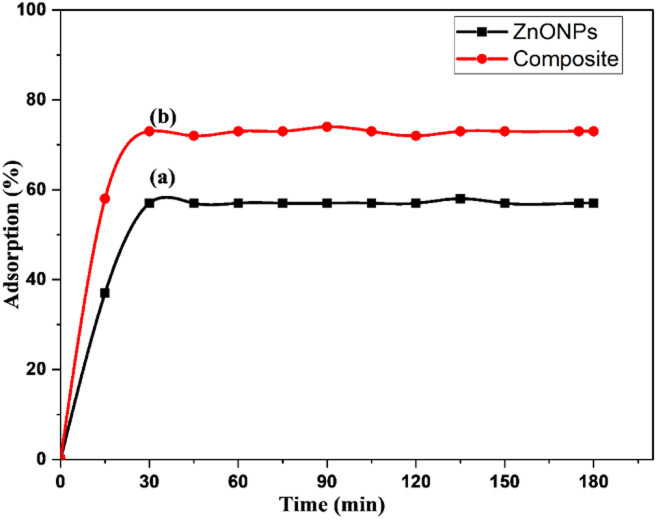
Adsorption–desorption equilibrium in the dark for: (a) ZnONPs and (b) ZnONPs@vitamin C adduct. Conditions: CR (50 ppm, 100 ml), catalyst = 0.05 g, time = 180 min.

Studies on the adsorption efficiency of ZnONPs and ZnONPs@vitamin C adduct were conducted in the dark for 180 min. The adsorption–desorption equilibrium was reached in 30 min (57% for ZnONPs, 73% for ZnONPs@vitamin C adduct) and remained constant throughout the experiment, as depicted in Fig. 17. This improvement in the adsorption capacity of the composite catalyst compared to ZnONPs was due to the positively charged surface of the composite, as proven by the zeta-potential values. The positively charged surface increased the attraction between the negatively charged CR dye particles (anionic dye) and positively charged catalysts in the case of the composite.

#### 3.3.3 Regeneration and reusability of the ZnONPs and ZnO@vitamin C adduct

Regeneration experiments were conducted to evaluate the catalytic tolerance of the ZnONPs and ZnONPs@vitamin C adduct. Initially, the degraded dye solutions were centrifuged, whereby the catalysts were isolated and then washed with a suitable ratio of water–ethanol (v/v; 1 : 1). The separated catalysts were dried in an oven.

**Fig. 18:**
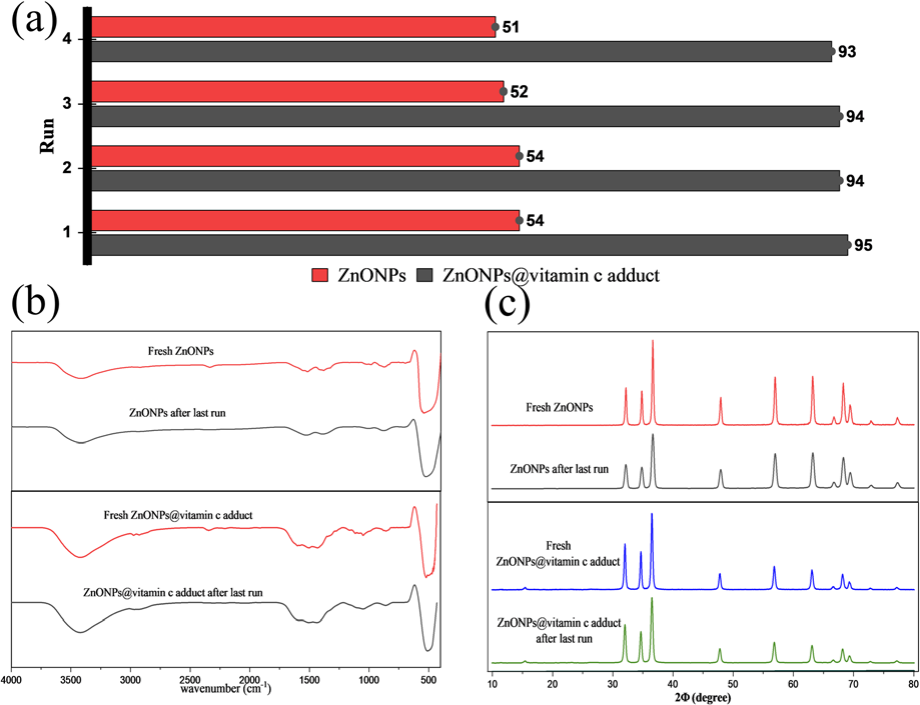
Regeneration and reusability evaluation as tested by: (a) catalytic degradation performance, (b) FT-IR spectra, and (c) XRD spectra of the catalysts.

The regenerated catalysts were evaluated for photocatalytic efficiency against CR dye degradation for four cycles. Fig. 18a shows the extent of tolerance of the regenerated catalysts; after the last run, the degradation performances were 93% and 51% for ZnONPs@vitamin C and ZnONPs, respectively. These results imply that the catalysts still have excellent degradation performance after four cycles. The identity and stability of the regenerated catalysts after the last run were emphasized by XRD and FT-IR spectra (Fig. 18b and c).

#### 3.3.4 Effects of the process parameters on the degradation of CR dye

##### 3.3.4.1 Amount of the catalyst (ZnONPs@vitamin C adduct)

**Fig. 19:**
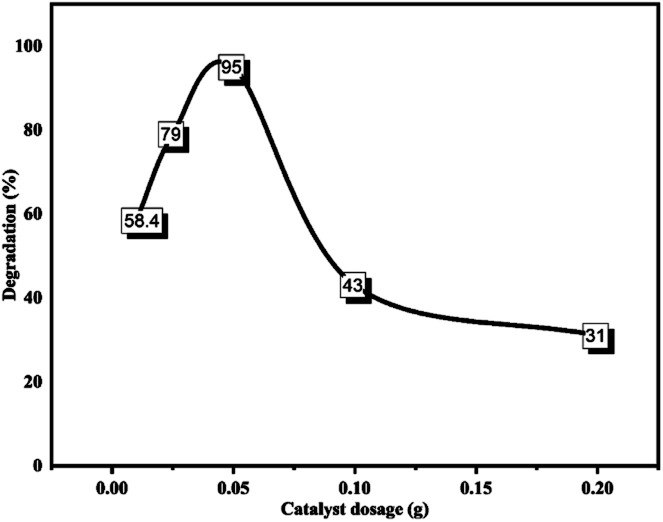
Effect of catalyst loading on CR photo degradation percentage. CR: 50 ppm, 100 ml; irradiation time: 180 min; pH of the medium: 7.

To obtain the optimized catalyst amount, various quantities (0.01, 0.025, 0.05, 0.1, and 0.2 g) of photoredox catalyst were studied keeping the other degradation parameters constant (see Fig. 19). After soaking in the dark, CR samples with various amounts of the ZnONPs@vitamin C adduct were irradiated for 180 min, and the results show that the optimum dosage of the catalyst was 0.05 g. Initially, the increase in catalyst amount from 0.01 to 0.05 g accelerated the degradation rate to reach a maximum rate of 58.4% to 95%, and then any excess of catalyst (*i.e.* more than 0.05 g) led to a considerable descent in the degradation percentage, which was due to the formation of an opacified suspension by the catalyst; also the percentage dye degradation was significantly affected by the photo-activated light penetration and catalyst surface active sites; whereby as the light penetration and photo-activated volume decreased, the active sites increase. The coincidence of activated molecules with ground-state molecules possibly led to the decrease in the degradation percentage at higher catalyst loading.^73^

##### 3.3.4.2 Effect of the initial dye concentration

Series concentrations of CR dye (25, 50, 75, and 100 ppm) were prepared to determine the influence of the initial dye concentration on the degradation process.

**Fig. 20:**
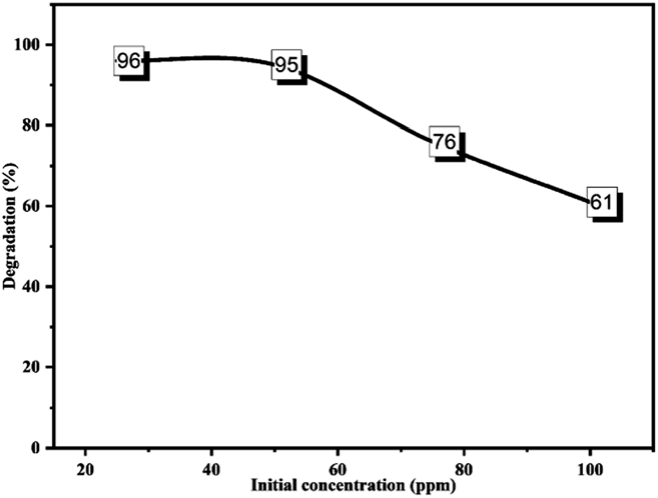
Effect of the initial concentration of CR on the photodegradation process. CR: 100 ml; catalyst: 0.05 g; irradiation time: 180 min; pH of the medium: 7.

Using the optimum amount of catalyst with CR solutions at 25 and 50 ppm, the maximum degradation (96% and 95%) was achieved. However, this progressively reduced as the CR concentration increased, as indicated in Fig. 20. The relation between the dye concentration and degradation percentage is well documented and fitted our outcomes.^74,75^ The degradation percentage is proportional to the generated ˙OH radicals on the surface of the nanocatalyst and the number of collisions between active species and the dye molecules. At high concentrations, the dye molecules quenched the active sites of the ZnONPs@vitamin C adduct, so the ˙OH radical production on the surface of the catalyst was suppressed, while a large portion of the irradiation light may also be absorbed by the dye molecules instead of the catalyst particles.^73,75^

##### 3.3.4.3 Effect of the pH

**Fig. 21:**
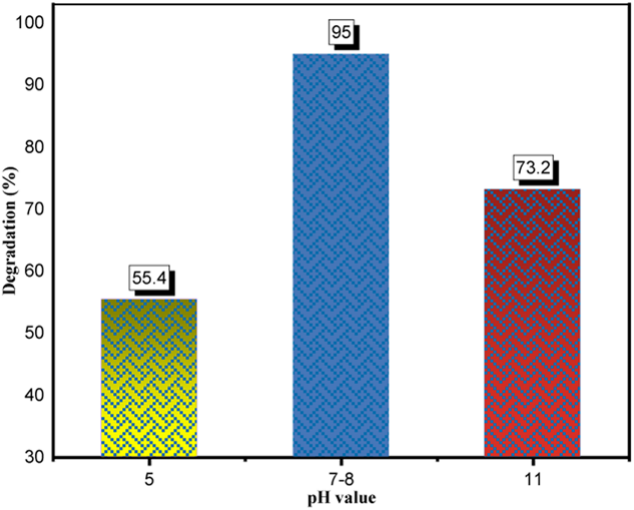
Effect of pH on the photodegradation process. CR: 50 ppm, 100 ml; catalyst: 0.05 g; irradiation time: 180 min.

In photodegradation, the pH alteration has a crucial impact on hydroxyl radical generation and significantly affects dye degradation processes.^15^ Here, the pH study of CR solution was performed under optimized conditions (0.05 g catalyst in 100 ml dye solution, 50 ppm, 180 min) as described in Fig. 21. Accordingly, under the optimal pH range of 7–8, the maximum dye degradation percentage (95%) was obtained. This experimental outcome could be due to two reasons: the zero-point charge (ZPC) and catalyst stability. Generally, the surface charge has a positive value under zero-point charge (pHzpc) and a negative value above (pHzpc). The zero-point charge of the ZnONPs was around 9.20.^76^ The zeta potential (see Fig. 14) of the developed catalyst (ZnONPs@vitamin C adduct) was positively charged (+9.7). Adsorption is a vital step in the photocatalytic degradation process. The electrostatic interaction between the positively charged surface of the ZnONPs@vitamin C adduct and the anionic CR dye resulted in high adsorption and increased hydroxyl radical formation. The other reason goes back to the stability of the catalyst (4) at various pH. Zinc oxide is generally insoluble in water but extremely soluble in acidic solutions (up to pH 4).^77^ In the pH range of 4–7, the ZnONPs were barely stable and partially soluble, so the efficiency of the catalyst (4) dropped down to 55.4% at pH 5. Moreover, the dye degradation was performed in basic media (pH 11) and it was relatively suppressed to 73.2%. In this situation, ZnONPs were negatively charged at a pH higher than 9.20 (ZPC); as a result, there was less interaction between the negatively charged dye and the catalyst.

##### 3.3.4.4 Effect of UV and solar light

**Fig. 22:**
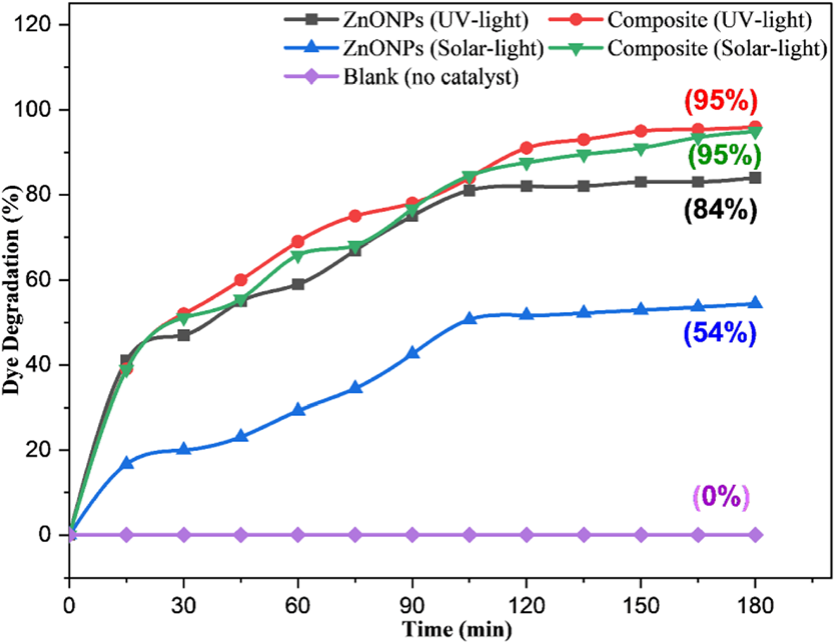
Photocatalytic degradation of CR (50 ppm) by pure ZnONPs (0.05 g) and the ZnONPs@vitamin C adduct (0.05 g) in the dark, and under UV and solar light irradiation, pH = 7–8.

Further investigations were performed to indicate the impact of UV and solar light irradiation on the catalytic degradation of CR dye. UV irradiation (180 min) of the dye solution (50 ppm) in the presence of the ZnONPs@vitamin C adduct (0.05 g) rather than solar light did not result a remarkable shift in the percentage of dye degradation (Fig. 22). This outcome reveals that UV light absorption did not significantly boost the generation of electron–hole pairs. However, a considerable boost in the photocatalytic activity of ZnONPs was observed in terms of the CR degradation percentage using the same condition. This outcome was due to the ZnONPs activity (*E*_gap_ = 3.2 eV) under UV light, which generated more electron–hole pairs and increased the percentage CR degradation (84%) compared to under the solar light (54%).

## 4. Comparison: prior data and current work

The degree of potency of the current catalyst (4) was compared to several previous works using ZnONPs as a catalyst (see Table 5).^74,78–87^ We were determined to demonstrate some important parameters (light source, dye concentration, time and percentage degradation). The information was extracted from the most recent works (entries: 1–10), which only focused on CR degradation by ZnONPs, and compared the works to the parameters in the present work (entry 11). The advantages of the prepared catalyst (ZnONPs@vitamin C adduct) in this work can be summarized in a few points: its activity under solar light instead of UV light, no need for a high power source, shortened irradiation time (180 min) compared to solar light (entry 6), and excellent degradation efficiency.

**Table 5:** Comparison of some literature data with the current work using ZnONPs for CR degradation

Entry	Type of catalyst	Irradiation source with intensity	Dye concentration	Time (min)	Degradation (%)
1 (ref. 78)	ZnONPs	UV light-30 W	30 ppm	60	90%
2 (ref. 83)	ZnONPs	UV-A lamps-365 nm	10 ppm	60	86.3%
3 (ref. 80)	ZnONPs	UV light-not specified	15 ppm	80	99%
4 (ref. 81)	ZnONPs	UV light-not specified	10 ppm	70	100%
5 (ref. 84)	ZnONPs	UV-vis light	10 ppm	140	97%
6 (ref. 85)	ZnONPs	Solar light	30 ppm	360	99%
7 (ref. 82)	(CZ-400)	UV-365 nm-125 W	100 ppm	120	65%
8 (ref. 86)	CS-ZnO	Sunlight	Not given	180	86%
9 (ref. 87)	C-ZnO	Sunlight	30 ppm	120	100%
10 (ref. 74)	Ag-ZnONPs	Visible light-24 W	50 ppm	180	94.2%
11	ZnONPs@vitamin C adduct	Solar light-15 W	50 ppm	180	95%
	ZnONPs	Solar light-15 W	50 ppm	180	54%

## 5. LC-MS study

**Fig. 23:**
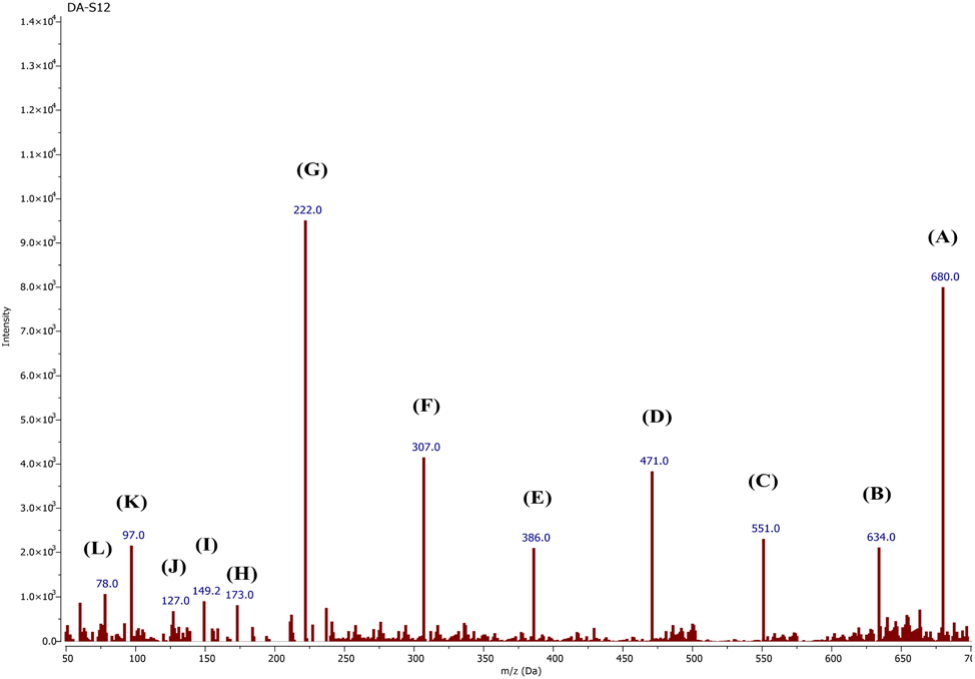
LC-MS spectrum of degraded CR dye solution after the entire dye color had vanished.

**Scheme 3:**
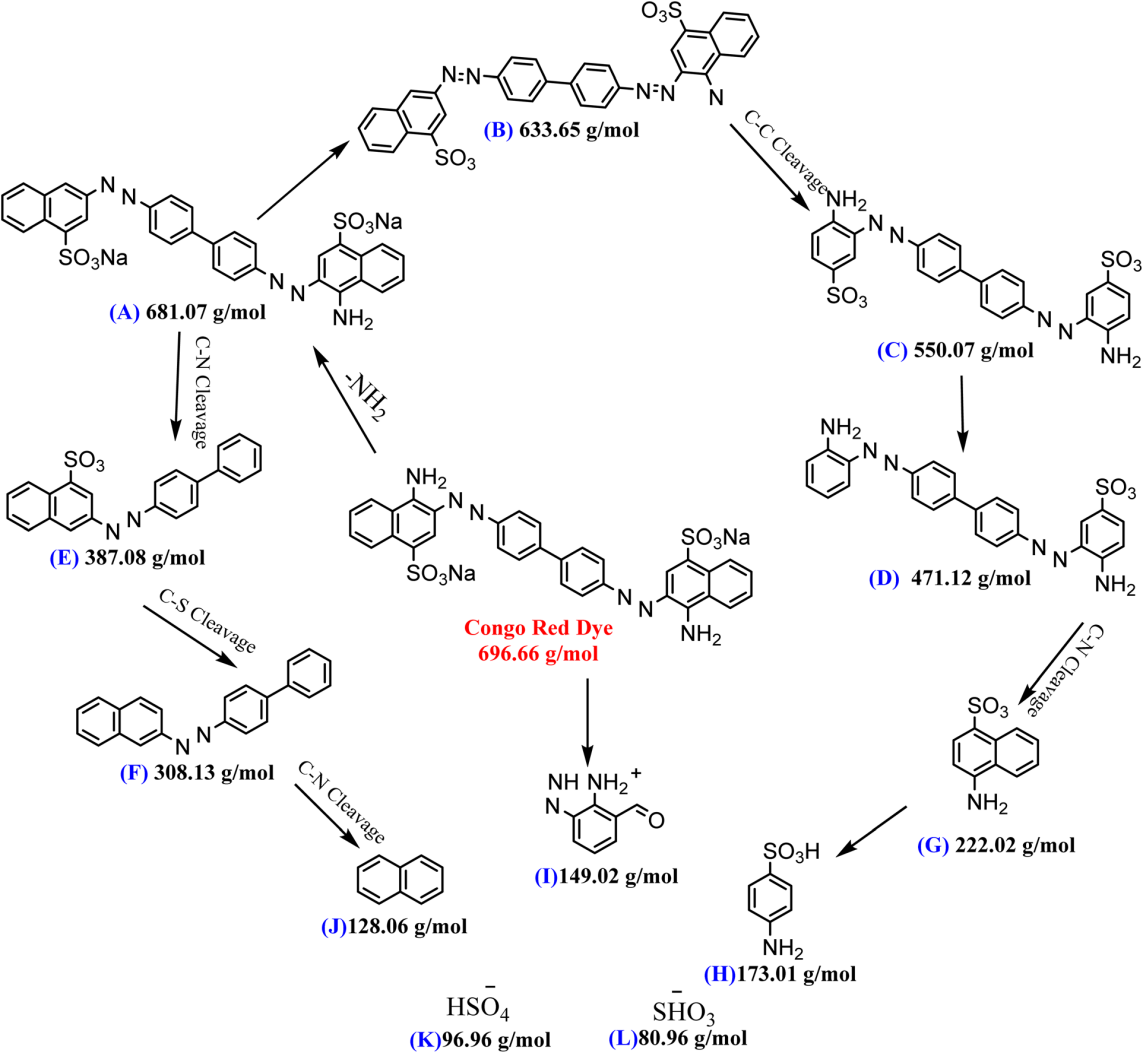
Possible products of the CR dye degradation by the ZnONPs@vitamin C adduct (4).

The fate of CR degradation by the nanocatalyst was studied using LC-MS. We were able to trap the degraded products by evaporating the degradable CR solution components and bombarding them with a beam of energy. The LC-MS spectrum (Fig. 23 and Scheme 3) illustrated the masses of the molecular ions (*m*/*z*) of the main fragments resulting from CR breakdown. The possible products were determined according to the weaker linkage cleavages, such as C–S, C–N, and –N=N–, in the CR molecule jointly with the LC-MS data. The data matched the information from the same dye degradation catalyzed by ZnONPs.^73,74^

## 6. Conclusions

This study aimed to fabricate a novel composite of ZnONPs@vitamin C adduct (4) by loading ZnONPs on to the surface of a substrate, a vitamin C adduct (3). Dramatically, the optical property and photocatalytic activity of the fabricated ZnONPs were improved through the coupling with the organic moiety (3). The identity of the fabricated composite (4) was fully characterized by various techniques. The IR spectra study revealed the chemical interactions between the surface of ZnO NPs and the l-ascorbic adduct (3) *via* the esteric and amidic carbonyl linkages. XRD study showed the existence of an amorphous composition in the crystalline structure of compound (4), which was due to the organic part (3). Elemental mapping and EDX studies confirmed the formation of compound (4). The study of the zeta-potential charge of ZnONPs and compound (4) illustrated the alteration of the zeta-potential value from −22.9 to +9.7, respectively, and showed the reasonable stability of the catalyst (4). In addition, the positively charged surface of the composite (4) facilitated a further catalytic improvement to adsorb the negatively charged CR (anionic dye). The results obtained from the UV-Vis-DRS spectrum analysis showed a decline in the optical band gap energy from 3.22 to 3.06 eV. The photoredox activity of ZnONPs and ZnO grafted on to adduct (3) was evaluated by breaking down CR dye at ambient temperature and in the presence of solar light. The photocatalytic degradation of compound (4) was extraordinarily raised (95%) compared to ZnONPs alone (54%). This outcome was confirmed by PL investigations; whereby the intensity of the PL absorption band was noticeably weaker for composite (4), which would boost the photocatalytic performance.

Studies on the stability, regeneration, reusability, catalyst amount, initial dye concentration, pH effect, and light source studies of the photocatalytic activity of both the synthesized ZnONPs@vitamin C adduct (4) and ZnONPs on CR dye degradation under solar light irradiation were conducted in depth. To make this catalyst available for commercial use, a comprehensive comparison was carried out between the ZnONPs that had been previously published and the data obtained from the herein developed composite (4). Under optimal conditions, the photodegradation of CR by the ZnONPs was 54% after 180 min of exposure to light, and it was 95% for the ZnONPs@l-ascorbic acid adduct after the same period. The PL study supported the hypothesis that the ZnONPs had enhanced photocatalytic performance. The components resulting from the photocatalytic degradation were determined using LC-MS spectrometry. Based on these results, the organic nanocomposite (4) will open a new way to treat pollution and serve as a potential photocatalyst for various applications.

## Author contributions

Dana A. Kader implemented the synthetic experiments and interpreted the experimental outcomes. Srood Omer Rashid wrote the manuscript and directed the project. Khalid M. Omer consulted in nanomaterial investigations.

## Conflicts of interest

The authors declare no conflict of interest.

## Supplementary Material

RA-013-D2RA06575D-s001
