# Supplementary material for: Green nanocomposite: fabrication, characterization, and photocatalytic application of vitamin C adduct-conjugated ZnO nanoparticles
Source: RSC Adv. 2023 Mar 29;13(15):9963–77. doi: 10.1039/d2ra06575d (PMC10050948; doi:10.1039/d2ra06575d)
Supplement: RA-013-D2RA06575D-s001 [file RA-013-D2RA06575D-s001.pdf]

Electronic Supplementary Material (ESI) for RSC Advances.
This journal is © The Royal Society of Chemistry 2023

Green Nanocomposite: Fabrication, Characterization, and Photocatalytic Application of Vitamin C Adduct Conjugated ZnO Nanoparticles

Dana A. Kader[a], Srood Omer Rashid[a,b*] and Khalid M. Omer[b]

a. Department of Chemistry, College of Education, University of Sulaimani, Kurdistan Region, Iraq.

E-mail: srood.rashid@univsul.edu.iq

b. Department of Chemistry, College of Science, University of Sulaimani, Kurdistan Region, Iraq.

Electronic Supplementary Information (ESI)

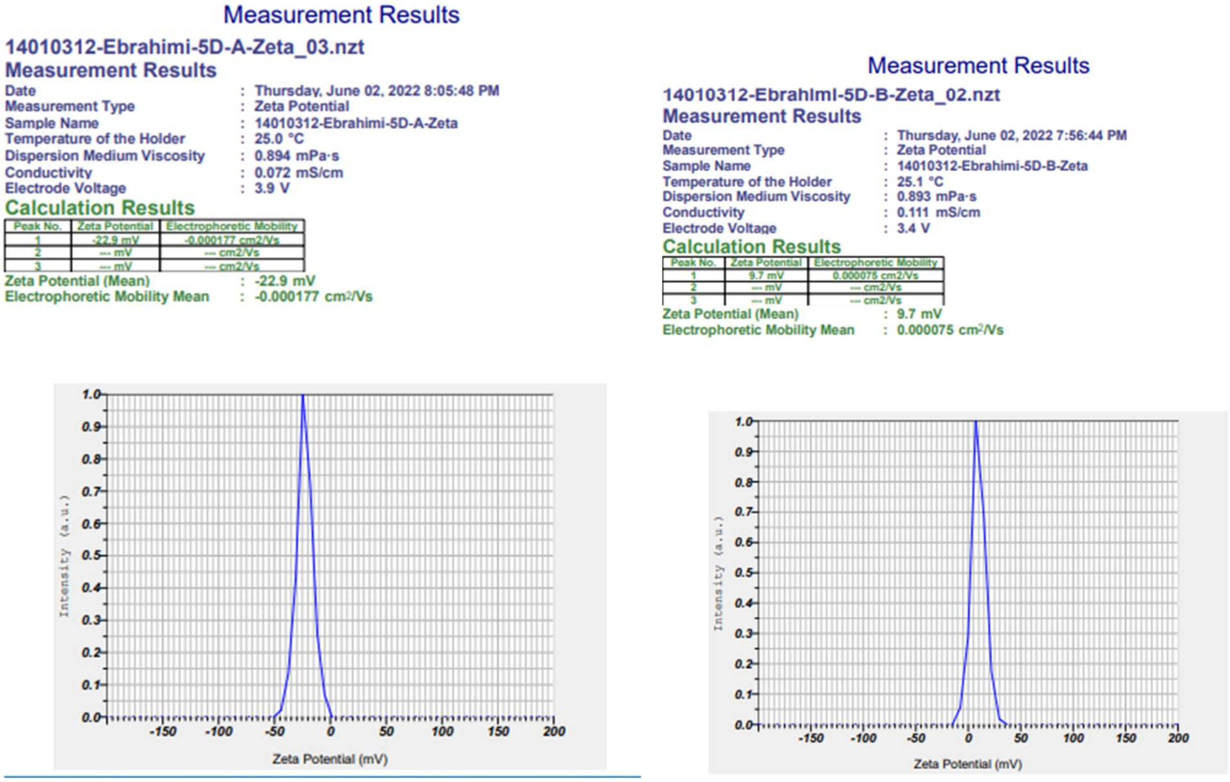

Fig. S1 Zeta potential value (-22.9) of pure ZnO NPs (a) and zeta potential value (+9.7) of the composite (b), respectively.

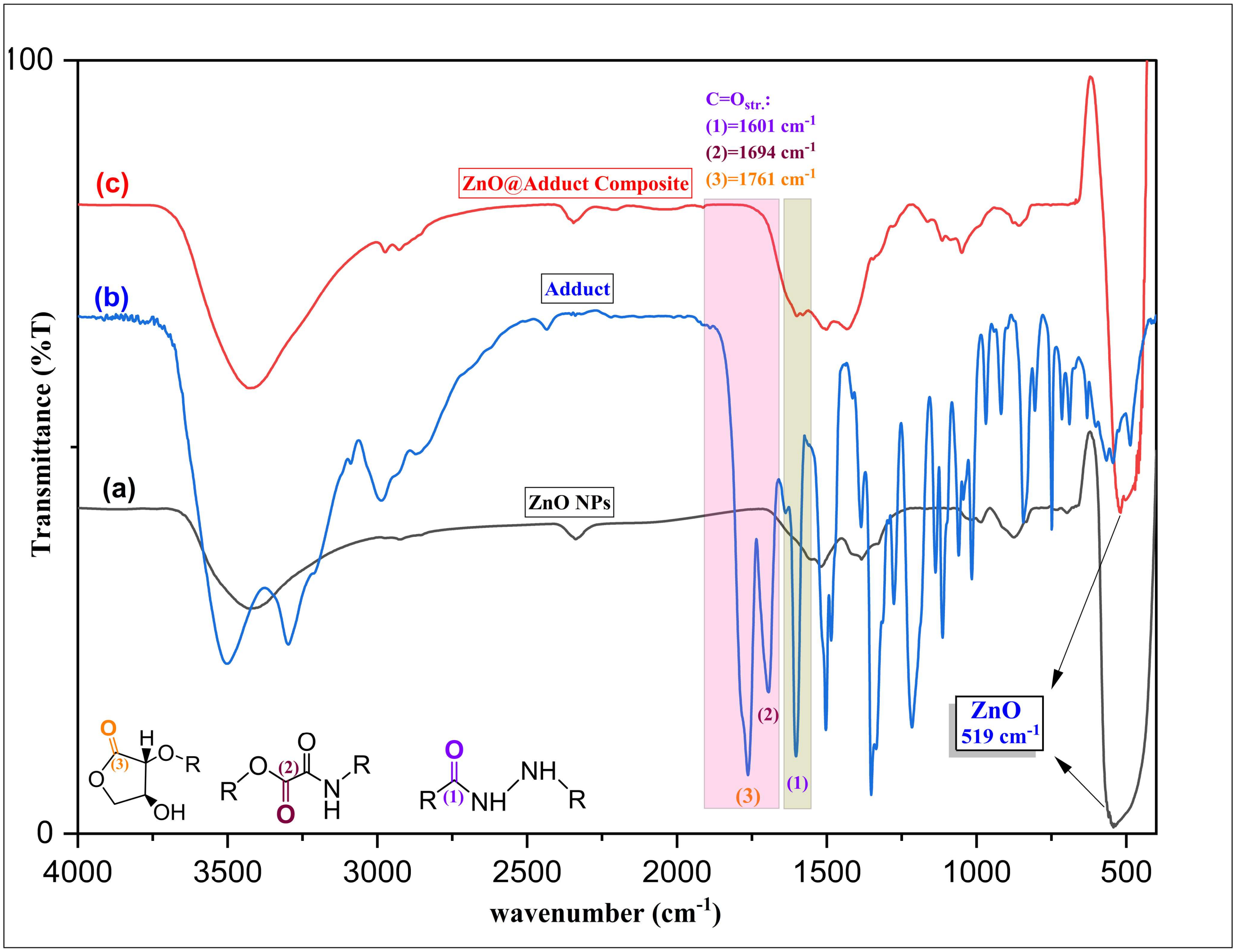

Fig. S2 FTIR spectra of (a) ZnONPs, (b) L-ascorbic acid adduct, and (c) L-ascorbic acid adduct conjugated ZnONPs, respectively.

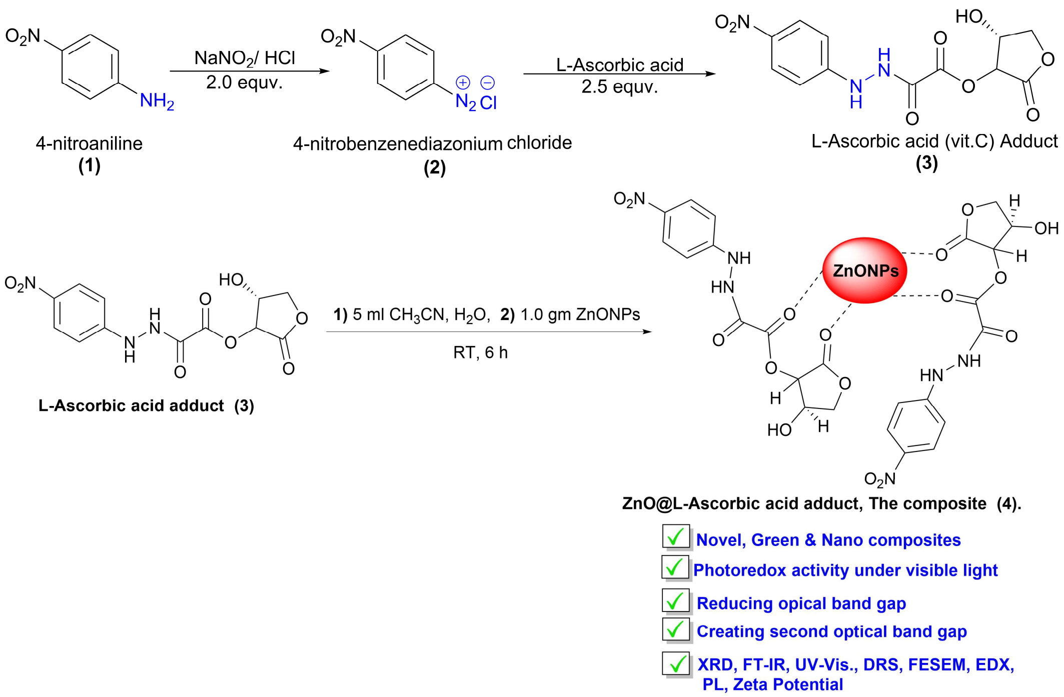

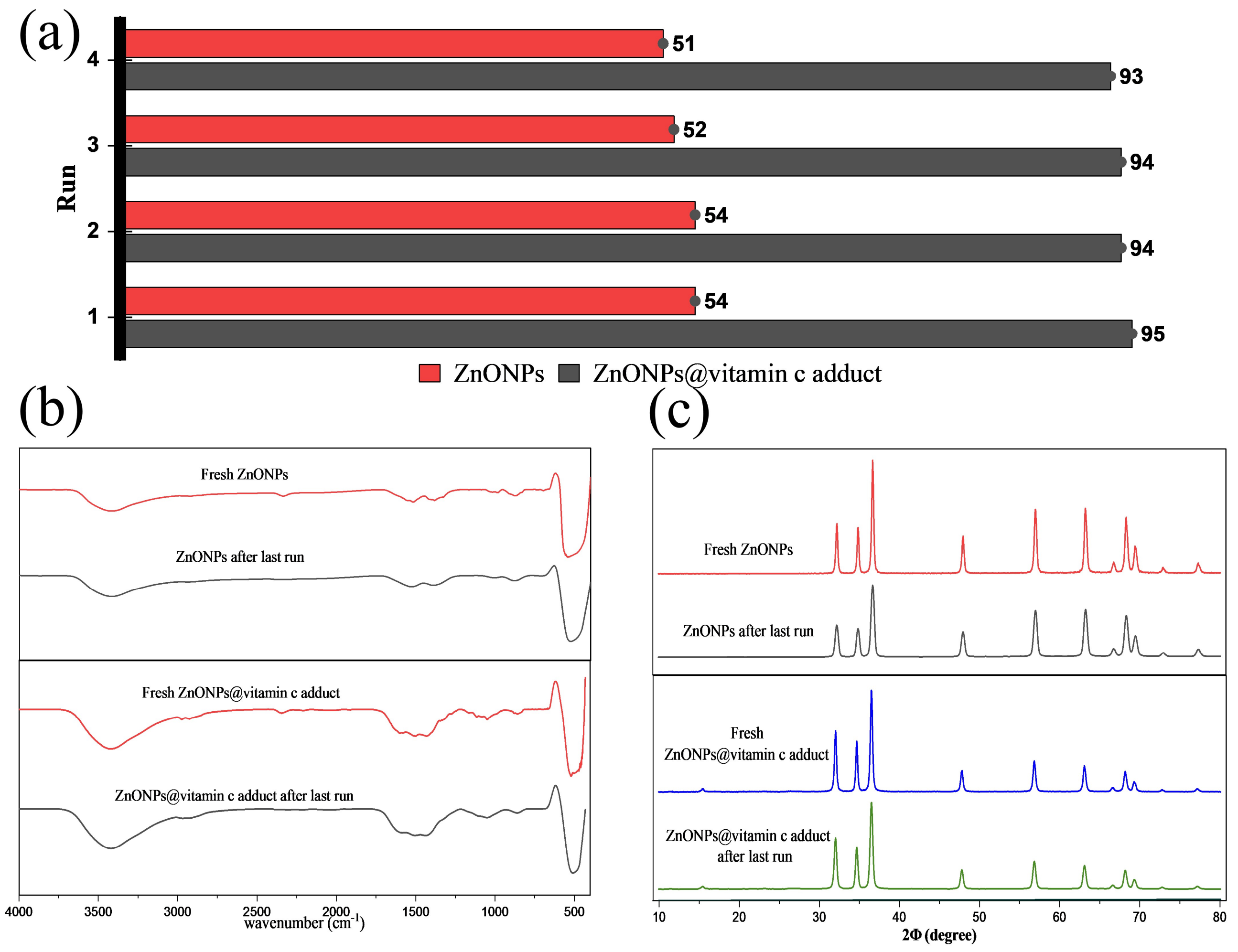

Fig. S3 Regeneration and reusability (a) Catalytic degradation performance, (b) FT-IR spectra and (c) XRD spectra of the catalysts.

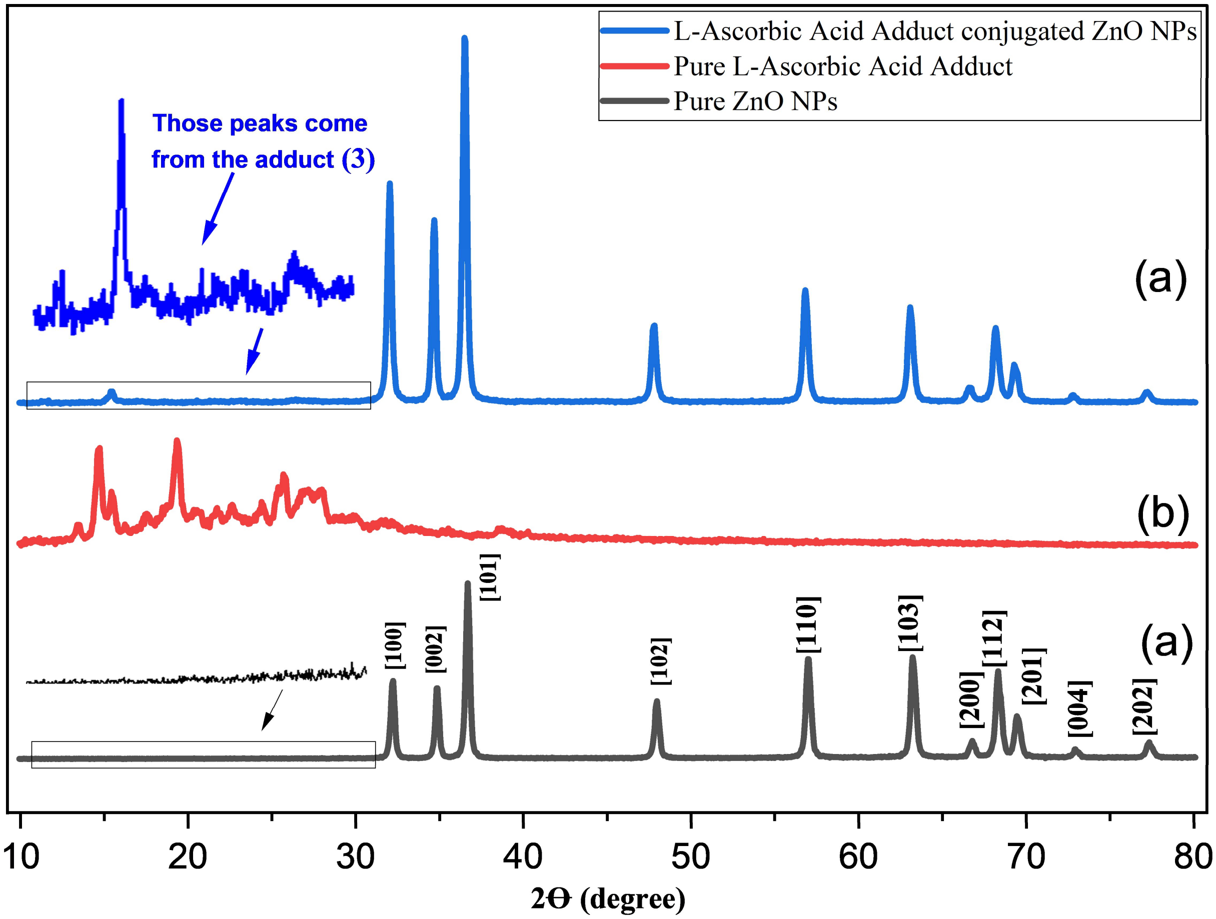

Fig. S4 XRD spectra of (a) ZnONPs (Black), (b) L-ascorbic acid adduct (Red), and (c)L-ascorbic acid adduct conjugated ZnONPs (Blue), respectively.

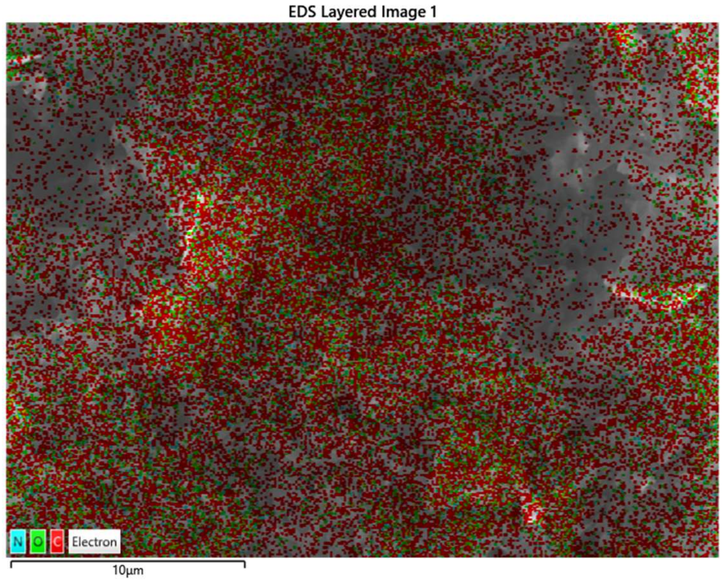

Fig. S5 EDX layered image of compound **(3)**

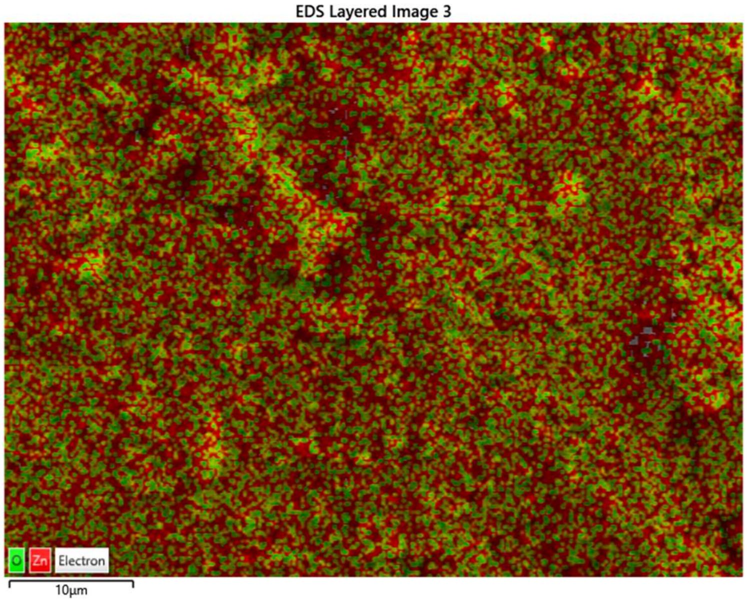

Fig. S6 EDX layered image of pure ZnO NPs

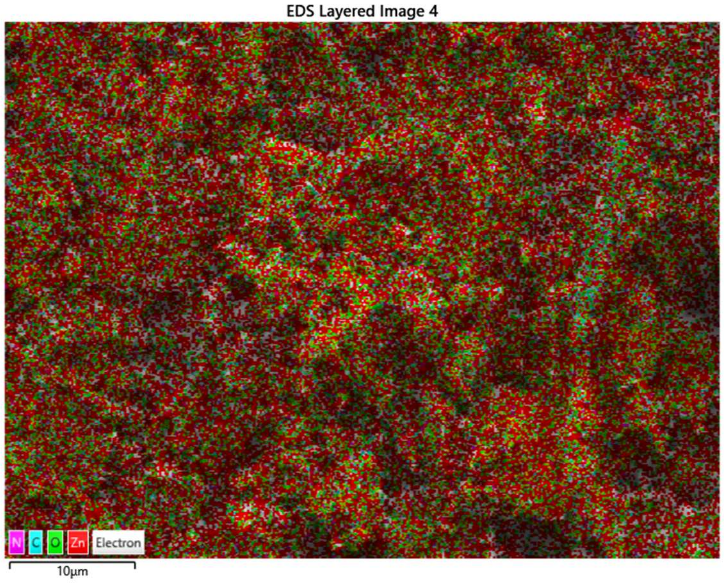

Fig. S7 EDX layered image of compound **(4)**

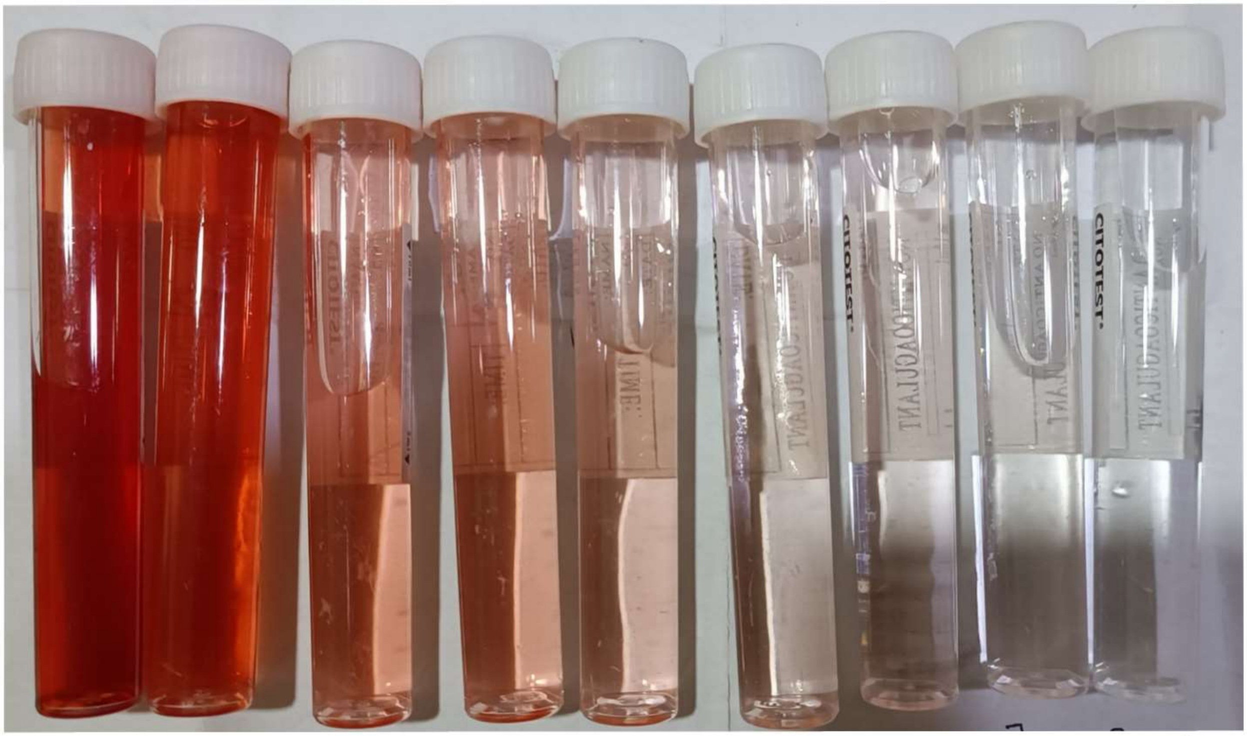

20 40 60 80 100 120 140 160 180 minutes

Fig. S8 photocatalytic degradation of Congo Red (CR)

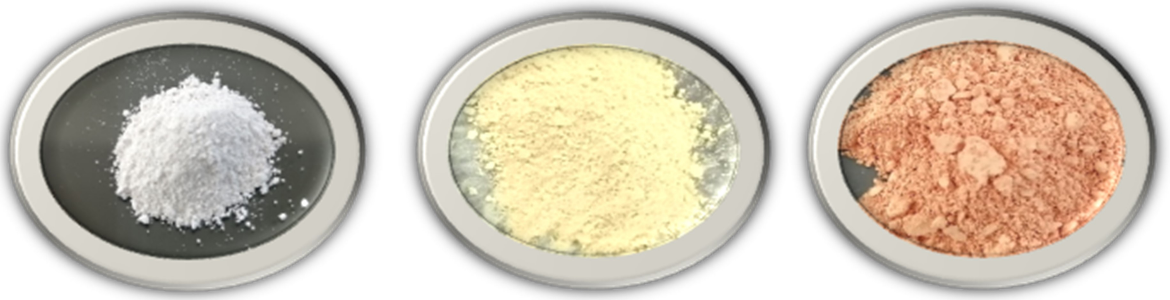

ZnONPs Vitamin C adduct **(3)** Composite**(4)**
ZnONPS@Vitamin C adduct

Fig. S9 Synthesized products
